# Barriers & Facilitators to Help‐Seeking Behaviour for Abnormal Lower Urinary Tract Symptoms in Men: Systematic Review

**DOI:** 10.1002/cam4.71214

**Published:** 2025-09-12

**Authors:** Stephen McIntosh, Bethany Harries, Matthew Perry, Mark Cropley, Bridget Dibb

**Affiliations:** ^1^ School of Psychology Faculty of Health and Medical Science, University of Surrey Guildford UK

**Keywords:** abnormal LUTS, bothersome LUTS, colorectal cancer, help‐seeking, prostate cancer, psycho‐oncology, testicular cancer

## Abstract

**Background:**

Men face numerous challenges when deciding whether to engage in help‐seeking for abnormal lower urinary tract symptoms (LUTS), with help‐seeking behaviour a multi‐step process.

**Objective:**

This systematic review explores the barriers and facilitators to help‐seeking behaviour in adult men for abnormal LUTS.

**Methods:**

Eight databases (PubMed, Web of Science, Cochrane Library, EBSCOhost, SCOPUS, OVID, ProQuest and PsychINFO) were searched between January 2023 and March 2024. Studies were required to meet the inclusion criteria informed by PRISMA guidelines. Qualitative and mixed‐method studies were included in the systematic review.

**Results:**

(*N*) = 17 full‐text articles were included in the review, totalling (*n*) = 704 participants. The systematic review uncovered four key themes: men have poor relationships with the healthcare system, some minority groups have dysfunctional cultural beliefs and attitudes towards help‐seeking behaviour, traditional gender views and perceptions of masculinity discourage help‐seeking behaviour and men have intrapersonal and external barriers.

**Conclusions:**

Men face considerable challenges when deciding to engage in help‐seeking for abnormal LUTS, with men generally lacking awareness and knowledge of what they should do when experiencing symptoms. This is more profound in men from minority groups. This review may have had a language bias as non‐English studies were excluded. This review may be essential to inform the development of interventions to facilitate help‐seeking behaviour for abnormal LUTS in men, specifically men from minority populations.

## 1 Introduction

Abnormal symptom of the lower urinary tract (LUTS), rectum and testicles are highly prevalent, as an estimated 44.7% of men experience abnormal LUTS worldwide [1], with the risk of said symptoms increasing with age [1, 2]. LUTS, rectal and testicular symptoms are often very worrisome for patients due to the possible severity of the symptoms, the difficulty in determining the cause of the problem and the negative impact on quality of life [3, 4].

Abnormal LUTS are defined as symptoms that impair the function of the prostate, urethra or bladder. They are categorised into urinary incontinence, storage/overactive bladder, voiding and postmicturition [5]. Abnormal symptoms affecting the scrotum and testicle include benign masses, infections, testicular torsions and testicular cancer [6]. Abnormal symptoms of the rectum vary from benign conditions such as haemorrhoids, anal fissures and rectal prolapse to serious conditions such as colorectal cancer [7]. Patients often delay help‐seeking for abnormal LUTS, rectal and testicular symptoms, but it is unclear as to why [8].

Due to the potential severity of abnormal LUTS, it is essential for men to engage in help‐seeking for abnormal symptoms [9]. Help‐seeking is a dynamic process whereby an individual acknowledges a problem and attends primary care for their problem [10]. It is important to determine the cause of the symptoms to rule out more severe problems. For example, urinary hesitancy is a symptom of both benign prostate hyperplasia (BPH) and prostate cancer (PCa; [11]), and prostate cancer is potentially life‐threatening, whereas BPH is not life‐threatening [11]. This example is one of many highlighting the importance of distinguishing the severity of abnormal LUTS, rectal or testicular symptoms. Existing literature in this area suggests that men do not readily engage in help‐seeking in primary care for abnormal LUTS, testicular and rectal symptoms [12]. Nevertheless, given the increasing number of studies in this area, a systematic review is essential to summarise and appraise the evidence from the existing literature.

This systematic review aims to fill the gaps in the literature by establishing the barriers and facilitators men encounter when deciding whether to access primary care when experiencing abnormal LUTS, rectal or testicular symptoms. The objective is to build on the paucity of existing literature with evidence‐based data on help‐seeking for LUTS, rectal and testicular symptoms, which may be applied by primary care and health policy to reduce the barriers men face when deciding to engage in help‐seeking.

## 2 Methodology

### 2.1 Search Strategy

Using PRISMA guidelines [13], Searches for papers were conducted between January 2023 and March 2024. No date restrictions were applied to the literature search. A search was carried out using the advanced search feature on several scientific databases. Searches were carried out using PubMed, Web of Science, Cochrane Library, EBSCOhost, SCOPUS, OVID, ProQuest and PsycINFO. Searches were carried out using the advanced search feature. The keywords and synonyms for the following search terms were categorised into: “Barriers & Facilitators”, “help‐seeking”, “abnormal lower urinary tract symptoms” and “male”. The search terms were connected using Boolean operators “AND” between categories and “OR” within categories. The search terms were derived from PubMed (Medical Subject Headings/MeSH), and a combination of these terms was used to produce an appropriate electronic search strategy.

The PubMed search items were linked to the acronym PICOS. The field code MeSH was used for searching items that had to be the subject of an article. For free search items that could be found in either the title or abstract of an article, the code [Title/Abstract] was used.

An asterisk symbol [*] was used for variation of words at once. The question mark symbol [?] was used as a wildcard to replace or denote an extra letter where spelling or word variation is possible (e.g., utili?ation). Filters were placed on the search only to include search results conducted on the human species, studies on adults, samples only consisting of men and studies using the English language. PsycINFO, Web of Science, Cochrane Library, EBSCOhost, SCOPUS, OVID and ProQuest handle different field codes for search items (see Appendix S1).

### 2.2 Study Selection

The inclusion criteria were developed using the PICOS (participants, intervention, comparator, outcome measure and study design) framework to assist with the selection of studies to be used in the review.

- Participants: Either diagnosed with genitourinary or colorectal cancer or men seeking help for new or persistent abnormal lower urinary tract symptoms
- Intervention: Qualitative studies exploring attitudes and beliefs towards help‐seeking behaviour for abnormal lower urinary tract, testicular and rectal symptoms in adult men.
- Comparator: Not available
- Outcome Measure: Attitudes, beliefs and experiences of help‐seeking that serve as barriers and facilitators related to engaging in help‐seeking behaviour (accessing primary care) for new/persistent abnormal lower urinary tract, testicular and rectal symptoms.
- Study Design: Qualitative studies and mixed‐method studies

To be included in the review, studies are also required to meet the following inclusion criteria:

- The sample population must be adults over the age of 18.
- Participants must be born and identify as male.
- Either diagnosed with a form of genitourinary cancer or colorectal cancer or seeking help for abnormal lower urinary tract symptoms.
- Help‐seeking is defined as seeking help from primary care services such as a GP or community pharmacist.

The first Author (SM) excluded research papers that featured sample populations that were not diagnosed with a form of genitourinary or colorectal cancer or were not seeking help for abnormal lower urinary tract symptoms; were not born male or identify as male; participants who were not adults, and the study was not a qualitative study. Following this, further eligibility inclusion was conducted by SM, who assessed the titles and abstracts of research papers against the inclusion criteria. Research papers in which the title and abstract met the inclusion criteria had a full‐text screening to assess eligibility further. Papers that were deemed eligible against the inclusion and exclusion criteria after the full‐text assessment had data extraction and quality assessment performed.

### 2.3 Title and Abstract Screening

The selected studies were quality assessed by the team members (SM and BH) using the revised version of the mixed‐methods appraisal tool (MMAT; [14]). The MMAT is a checklist with questions to assess the methodological quality of research using qualitative, quantitative randomised controlled trials, quantitative non‐randomised, quantitative descriptive and mixed‐method studies. As suggested by the authors of the MMAT, team members (SM and BH) independently appraised the selected studies.

The MMAT was used to determine the methodological quality of the articles in the criteria in the category of the studies design. Following the guidance from the authors of the MMAT tool, studies responding as ‘No’ or ‘Can't tell’ to the screening questions would not be considered empirical research and would be excluded. For the remaining articles, five yes‐no questions appraised the methodological quality of the articles. If all five answers were ‘Yes’, the study was included in the review. Studies with at least one ‘No’ or ‘Can't tell’ were discussed by the research team to decide whether to include them in the review. Consequently, the studies included in the review were rated as good quality by the MMAT appraisal tool (see Appendix S2).

For each study selected to be included in the review, team members (SM and BH) extracted data using a previously tested standardised form. Author (SM) initially extracted data using a previously tested standardised form. The second author (BH) performed the independent data extraction on 20% of the selected studies. Data collection was (a) general study information (authors, study year, country of origin, enrolment period, inclusion and exclusion criteria); (b) population details (ethnicity, sample size and age); (c) details of the study outcomes (attitudes, beliefs and knowledge) towards abnormal lower urinary tract symptoms (LUTS), PCa, testicular cancer (TCa) and colorectal cancer (CRC) testing; (d) outcomes and findings of the papers (see Appendix S3).

### 2.4 Data Synthesis

Qualitative data was analysed and integrated using narrative synthesis following the framework set out by Popay et al. [15]. (1) Data was organised and summarised across studies. (2) Key data was grouped to form a preliminary synthesis to identify patterns in the data. (3) Relationships within and across the data in the included studies were explored. (4) The robustness of the included studies was assessed in terms of their study quality and consistency.

## 3 Evidence

### 3.1 Description of Studies

**FIGURE 1:**
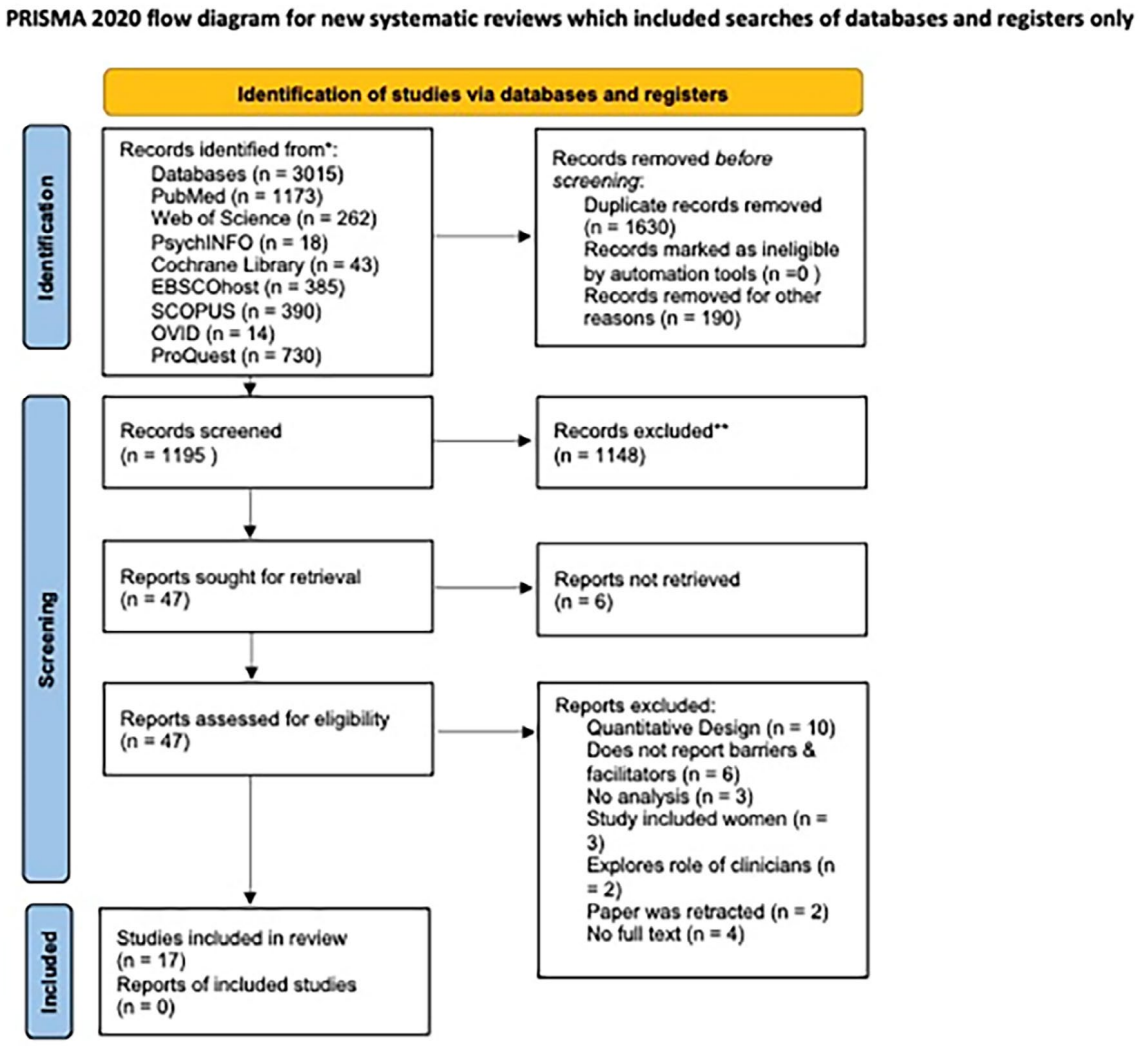
PRISMA flow diagram.

A total of 3015 studies were identified through a literature search. Among them, 2968 studies were excluded as either duplicates or not meeting the inclusion/exclusion criteria after screening. A total of 47 studies underwent full‐text screening for eligibility. After full‐text screening, a further 30 studies were excluded. Reasons for exclusion were that the papers did not report the barriers and facilitators, did not conduct analysis, the study analysed perceptions and performance of the screening methods, no full text was available and the sample population included women and healthcare professionals (see Figure 1). Subsequently, 17 studies met the inclusion criteria and underwent data extraction. See Figure S1 in the Appendix. A narrative synthesis was performed to analyse the studies included in the review.

The studies included in the review were a mixture of qualitative (*n* = 15; [16, 17, 18, 19, 20, 21, 22, 23, 24, 25, 26, 27, 28, 29, 30]) and mixed‐method studies (*n* = 2; [31, 32]). The sample sizes ranged between (*n* = 135) and (*n* = 9) with a total of (*n* = 701) participants across all 17 studies. Several studies reported participant nationality, whilst others reported participant ethnicity. The sample population of the studies consists of a diverse range of groups; (*n* = 4) Caucasian men, (*n* = 6) African American men, (*n* = 3) Afro‐Caribbean men, (*n* = 1) South East Asian men, (*n* = 1) Arab American men and (*n* = 2) West African men. Of these studies, (*n* = 10) were men from a minority ethnic group in the country where the study was conducted. Abnormal LUTS can comprise several diseases; hence, there is a variation in the diseases included in the studies. Of the included studies, (*n* = 1) explores the barriers and facilitators to help‐seeking for TCa (*n* = 14) explore PCa and (*n* = 2) explore CRC (see Table 1 for summary of papers).

**TABLE 1:** Showing summary of findings from papers included in the review.

Authors	Year	Country	Data collection method	Cancer type	Population	Main findings
Oliver	2007	United States	Qualitative interviews	Prostate Cancer	African American men in rural Alabama	African American men do not trust the healthcare system due to historical malpractice. African American men also lack an understanding of prostate cancer and prostate cancer screening methods
Conde et al.	2011	United States	Qualitative interviews	Prostate Cancer	Filipino men in Hawai’i	Filipino men lack awareness of knowledge and awareness of prostate cancer. Hold negative beliefs towards prostate cancer, are fearful of being diagnosed with prostate cancer and present in primary care at a later stage
Earl et al.	2022	United States	Mixed Methods	Colorectal Cancer	African American men from Atlanta	African American men lack knowledge of colorectal cancer, cultural beliefs discourage attending primary care and machoism contributes to delay. However, men want culturally sensitive awareness programmes
Shungu and Sterba	2021	United States	Qualitative interviews	Prostate Cancer	African American men from South Carolina	African American Men have poor relationships with the healthcare system and lack trust with HCPs. Men also lack knowledge of prostate cancer, lack access to services and are fearful of cancer diagnosis. However, men want culturally sensitive awareness programmes
Medina‐Perucha et al.	2017	United Kingdom	Qualitative Interviews	Prostate Cancer	Men from the United Kingdom	Perceptions of masculinity contribute to delay. Men are also fearful and embarrassed of prostate cancer screening methods. Nevertheless, previous health problems facilitate future help‐seeking behaviour
King‐Okoye et al.	2019	Trinidad & Tobago	Qualitative Interviews	Prostate Cancer	Men from Trinidad and Tobago	Trinidadian men have dysfunctional beliefs about prostate cancer and have taboo over the symptoms and testing methods for prostate cancer. Men also lack knowledge of prostate cancer testing and seek alternatives to medical treatment
Woods et al.	2004	United States	Mixed Methods	Prostate Cancer	Black men from Southern California	Black mAusen lack knowledge of prostate cancer, have poor communication with HCPs, lack trust of HCPs and perceive testing for prostate cancer as a threat to masculinity. Men want the healthcare system to re‐build trust and build a relationship with the community
Seymour‐Smith et al.	2016	United Kingdom	Qualitative Interviews	Prostate Cancer	Caribbean men from the United Kingdom	Cultural beliefs in the United Kingdom discourage help‐seeking behaviour. Caribbean men have negative attitudes towards testing and Caribbean men perceive that the testing methods for prostate cancer are sexual acts
Ocho & Green	2013	Trinidad & Tobago	Qualitative Interviews	Prostate Cancer	Men from Trinidad and Tobago	Men lack awareness of prostate cancer, have negative views towards prostate cancer testing and perceive prostate cancer testing as a threat to masculinity. Nevertheless, men will go to the doctor with “red flag” symptoms and have a preference for PSA testing
Alsayid et al.	2019	United States	Qualitative Interviews	Colorectal Cancer	Arab American men from San Francisco and Massachusetts	Arab American men lack trust in HCP and lack trust in modern medicine. Men also dislike screening methods for colorectal cancer and have poor access to healthcare services. Men would prefer alternative testing methods and improve their access to healthcare services
Ettridge et al.	2018	Australia	Qualitative Interviews	Prostate Cancer	Australian men	Australian Men have dysfunctional beliefs towards prostate cancer, hold stigma towards disease and have poor social support networks. Stronger social support networks would facilitate help‐seeking behaviour
Enaworu and Khutan	2016	Nigeria	Qualitative Interviews	Prostate Cancer	Nigerian men in Nigeria	Nigerian men have poor knowledge of prostate cancer, lack access to services. Men would be more likely to go to the doctor if they had stronger support networks, improved access to services and improved knowledge of prostate cancer
Saab et al.	2018	Ireland	Qualitative Interviews	Testicular Cancer	Men from the Republic of Ireland	Men had poor knowledge of symptoms affecting the testicles and have poor knowledge of HCPs. Men avoid going to the doctor to preserve masculinity. However, homosexual men reported being more inclined to access primary care than heterosexual men
Ezenwankwo et al.	2021	Nigeria	Qualitative Interviews	Prostate Cancer	Men from Nigeria	Men lack awareness of prostate cancer and have difficulties accessing health services. Nigerian culture discourages from going to the doctor for prostate cancer and shares cancer fatalism. Nigerian men reported they will go to the doctor if they have “severe” symptoms
Fyffe et al.	2008	United States	Qualitative Interviews	Colorectal Cancer and Prostate Cancer	African American men from New Jersey	Men have poor communication with HCPs and lack trust in the healthcare system. Men delay going to the doctor as the DRE creates embarrassment and some men cannot afford to go to the doctor. Men want targeted outreach to African American communities
Forrester‐Anderson	2005	United States	Qualitative Interviews	Prostate Cancer	African American men from the Baltimore Metro area	Men have poor knowledge of prostate cancer, fear testing for prostate cancer and are embarrassed by testing for prostate cancer. Cultural beliefs discourage men from going to the doctor. Men also distrust the government, healthcare system and have poor communication with HCPs. Men want targeted outreach to African American communities

Eight descriptive themes emerged as barriers and facilitators across the papers. They fall into the following groups: (1) distrust of the healthcare system; (2) re‐building relationships with the healthcare system; (3) cultural stigma and attitudes; (4) community outreach programmes; (5) external barriers; (6) presence of symptoms; (7) extraneous barriers; (8) extraneous facilitators. Four overarching themes were developed: relationships with the healthcare system, cultural beliefs and attitudes, masculine beliefs and intrapersonal and external barriers (for a summary of papers included in the paper, see the table below; for full data extraction, see the Appendix).

### 3.2 Relationships With the Healthcare System

The relationship that one holds with the healthcare system plays an important role in whether individuals decide to engage in help‐seeking behaviour for abnormal LUTS. This theme is comprised of three sub‐themes: distrust of the healthcare system, poor communication with HCPs and positive relationships with HCPs. The analysis revealed that whilst poor relationships with the healthcare system and healthcare professionals are barriers to help‐seeking, building relationships with HCPs and healing wounds between the care system facilitates improved knowledge and improved informed decision making (IDM) to engage in help‐seeking for abnormal symptoms.

In eight of the seventeen studies, men reported a distrust of the medical care that they will receive when seeking help for abnormal LUTS [16, 20, 21, 22, 24, 27, 30, 31]. This barrier was most prevalent in men from minority ethnic groups. Six of the seven studies that reported distrust of the healthcare system took place in the United States [16, 21, 22, 27, 30, 31].

Beliefs held by African American men contributing to the lack of trust in the healthcare system stem from historical medical malpractice towards African Americans, notably the Tuskegee Syphilis experiment [22, 27, 30]. Subsequently, due to the lack of trust, African American men report having a culture with a shared reluctance to avoid going to the doctor [21, 22, 27, 30, 31]. Moreover, there is a shared perception among African American men in the United States that they do not receive equal treatment compared to white Americans [21, 31]. Consequently, some African American men share the belief that HCPs have ulterior motives; hence, they will not be open when discussing PCa with HCPs [30].

Three further studies reported a distrust of healthcare and a distrust of medicine and practitioners [16, 20, 22, 24]. Some Afro‐Caribbean and West African men lack trust in the diagnostic ability of doctors and seek alternative treatments for PCa through witch doctors and herbal medicines and remedies [20, 24]. This belief differs from that of some Arab American men who do not engage in help‐seeking for CRC as they believe that cancer is a made‐up concept by pharmaceutical companies to profit from the sale of drugs [16]. Furthermore, some men reported a preference to have consultations with senior doctors as they lack trust in the diagnostic ability and medical knowledge of junior doctors [22].

Poor communication with HCPs was reported by four of the seventeen studies included in the review [16, 17, 20, 32]. Poor communication was a barrier more commonly reported by men from minority ethnic groups [16, 17, 32]. Participants reported that primary care physicians were often too busy to arrange appointments, and when an appointment was arranged, some GPs did not have enough time for their patients during the consultations [16, 17, 20]. Two studies found that GPs did not fully explain the potential risks of the PSA test, inform African American men why they should or should not be offered a PSA test, or explain why they had a DRE [21, 32]. Men reported similar experiences with CRC, as men reported that GPs did not provide enough information about CRC screening and rushed consultations [16].

#### 3.2.1 Improved Relationships With Healthcare Professionals

Across the reviewed papers, it was widely reported that healthcare providers and healthcare professionals play an important role in facilitative engagement in help‐seeking for abnormal LUTS. Eight of the seventeen papers reported that healthcare professionals serve as facilitators of help‐seeking behaviour [16, 17, 20, 26, 27, 30, 32]. Participants often reported wanting to know the reasons behind particular events and processes and reported that clinicians should provide them with information to aid IDM in men across all groups for engaging in help‐seeking for abnormal LUTS [30, 32]. Men from minority groups report that healthcare providers have a duty to mend strained relationships [27, 30]. Nevertheless, men across all groups shared the belief that improved relationships with GPs and improved access to healthcare services will encourage men to engage in help‐seeking for abnormal LUTS and CRC [16, 17, 20, 27, 30].

### 3.3 Cultural Beliefs and Attitudes

Cultural beliefs and norms play a key role in determining whether an individual will engage in help‐seeking for abnormal LUTS. Stigma towards engaging in help‐seeking for abnormal LUTS is often negative and was a common theme to emerge from the reviewed papers. Eleven of the seventeen papers reported negative cultural beliefs and stigma towards engaging in help‐seeking for abnormal LUTS [16, 17, 19, 20, 21, 22, 24, 26, 27, 29, 31]. This theme is comprised of two sub‐themes: negative cultural beliefs towards help‐seeking for LUTS and community outreach programmes.

#### 3.3.1 Negative Cultural Beliefs Towards Help‐Seeking for Abnormal LUTS

Negative cultural beliefs were prominent among the included papers, with eight of the included studies reporting cultural stigma towards engaging in help‐seeking for abnormal LUTS [17, 20, 21, 22, 24, 27, 31]. Afro‐Caribbean men reported that it is a cultural norm to delay engaging in help‐seeking for problems that may impact sexual function and have a preference for over‐the‐counter medication before seeking help from primary care physicians [24]. Beliefs about the causes of PCa and abnormal LUTS in West African culture deter some men from engaging in help‐seeking, as cultural norms imply that PCa is caused by witchcraft, being unfaithful to partners and having PCa will make one unfit to participate in religious rituals or ceremonies [20]. This cultural norm differs from that of African American culture, which discourages help‐seeking in primary care due to historical malpractice [27, 31].

Cultural fatalistic beliefs towards cancer can create delay towards help‐seeking within some cultures. Some Filipinos and African American men delay engaging in help‐seeking for abnormal LUTS as it is a cultural norm to delay help‐seeking due to fatalistic attitudes towards cancer held by many within the culture [17, 21].

Cultural views held against screening methods, particularly the colonoscopy and the DRE, act as barriers towards engaging in help‐seeking [22, 25, 26, 29]. Caribbean men reported that cultural views serve as barriers to help‐seeking [26, 29]. The DRE is stigmatised among some Caribbean men as the performance of the DRE is seen as a sexual act. This cultural belief is shared among some African American men who view the DRE as a sexual act [21, 22]. Nevertheless, the negative cultural views towards homosexuality held in the Caribbean make some men very reluctant to engage in help‐seeking [26, 29].

#### 3.3.2 Challenging Negative Beliefs and Stigma Towards Help‐Seeking

Challenging cultural views and stigma towards help‐seeking can facilitate help‐seeking in primary care for abnormal LUTS. Nevertheless, it is important to note that methods to challenge views and stigma are culture‐specific. Afro‐Caribbean men from two papers reported that the presence of community outreach programmes targeted specifically for the Afro‐Caribbean community would reduce stigma towards screening methods, leading to an uptake of PCa screening [24, 29]. Re‐building trust through community outreach and improved relationships with clinicians will challenge the culture of distrust of the medical system in the United States held by many African American men [21, 22, 27]. Filipino men report that similar action can be taken to dispel negative cultural attitudes towards help‐seeking through improving relationships with clinicians to facilitate IDM [17].

### 3.4 Traditional Gender Views

Self‐perceptions of masculinity and machoism play a key role in men's decisions to engage in help‐seeking for abnormal LUTS. The perceptions of one's masculinity are a prominent barrier to help‐seeking for abnormal LUTS across ages and cultures, with eight of the seventeen reviewed studies reporting that the preservation of masculinity is a barrier to help‐seeking [17, 22, 24, 25, 26, 27, 28, 30]. Moreover, due to the perceived trivialisation of men's health problems, men delay attending primary care when experiencing abnormal symptoms due to a fear of being labelled a hypochondriac [28]. Whilst the preservation of masculinity is a barrier to help‐seeking, the presence of “red flag” symptoms prompt men to engage in help‐seeking for abnormal LUTS [18, 24, 26, 32].

#### 3.4.1 Masculinity and Machoism

Men generally believed in adherence to traditional gender expectations, such as exhibiting restricted emotional expression and a need for independence not burdening others with problems and minimising the severity of symptoms to maintain their perception of masculinity [25, 30]. Younger men exhibited stoic attitudes towards abnormal LUTS to maintain their masculinity [28]. Whereas, among older Afro‐Caribbean men, abnormal LUTS symptoms are normalised as “part of the natural ageing process” [24], resulting in older Afro‐Caribbean men typically engaging in delayed help‐seeking for abnormal LUTS due to the perception that symptoms are expected when becoming an older man [24].

Screening methods for abnormal LUTS act as a barrier as men perceive screening as emasculating [16, 17, 22, 25, 26, 27]. Men delay screening for PCa due to the stigma surrounding the DRE, as men report fear of performing the procedure as it takes away one's manhood [22, 27]. Men have different views on who should perform the DRE, as Filipino men report great embarrassment in performing the DRE when a female nurse is present, whilst Afro‐Caribbean men have a preference for female nurses as they are less of a challenge to their manhood [17, 26].

### 3.5 Presence of Symptoms

Symptoms affecting perceived masculinity and “red flag” symptoms facilitate help‐seeking regardless of the negative stigma, embarrassment of screening or fear of screening [18, 24, 26, 28, 32]. Men will engage in timely help‐seeking for “red flag” symptoms such as blood in urine and urine retention as they are perceived with extreme severity [24]. Symptoms affecting sexual function facilitate timely help‐seeking due to the perceived emasculation due to the loss of manhood [19, 32]. Hence, receiving treatment for symptoms affecting sexual function is viewed as a restoration of one's manhood [32]. However, some African American men reported that they will continue to delay engaging in help‐seeking due to a fear of finding out the truth and the belief that African Americans are inferior, so there is no point attending primary care regardless of the severity of symptoms [21].

### 3.6 Intrapersonal and External Barriers to Help‐Seeking Behaviour

Men face considerable intrapersonal and external barriers to engaging in help‐seeking for abnormal LUTS. Men reported poor awareness of symptoms causing abnormal LUTS and a lack of awareness of how to access healthcare, whilst some men also reported a lack of access to healthcare services and a lack of culturally diverse material. Fourteen studies reported both intrapersonal and external barriers to help‐seeking [16, 17, 18, 19, 20, 21, 22, 23, 25, 27, 28, 30, 31, 32]. However, men from six studies reported what can be done to mitigate the external barriers to encourage help‐seeking for abnormal LUTS [16, 22, 25, 26, 27, 31].

#### 3.6.1 Intrapersonal Barriers to Help‐Seeking for Abnormal LUTS

Men across all groups report an overall lack of awareness of men's health problems and what to do when experiencing symptoms affecting the lower urinary system [17, 20, 21, 27, 28, 32]. Young men lack knowledge of their genital area, lack knowledge and awareness of TCa and report that the trivialisation of men's health problems contributes to delays in engaging in help‐seeking for abnormal testicular symptoms [28]. Men lack awareness of CRC screening methods, with African American and Arab American men reporting very limited knowledge of CRC screening methods but reporting better knowledge of screening ranges [16, 31]. Similar findings were observed for PCa, as men report a lack of knowledge of PCa but also report a lack of available information about PCa and PCa screening [17, 19, 21, 27].

Men delay accessing healthcare due to fear of the screening methods and fear of receiving a positive diagnosis [17, 21, 22, 25, 27, 28, 30]. Fear is consistent among age groups, with both younger and older men reporting a delay in help‐seeking due to fear of being diagnosed with a potentially serious health condition [17, 28]. However, men report being very fearful of CRC screening and PCa screening (specifically the DRE) and will delay engaging in help‐seeking to avoid the procedures [21, 22, 25, 27, 30].

#### 3.6.2 External Barriers to Help‐Seeking

Access to healthcare services is a key barrier to accessing primary care, as men who do not have access to healthcare either cannot seek help from primary care or will find it extremely difficult to attend primary care [16, 17, 20]. Men with lower incomes report that they cannot afford to pay for screening or consultations, and their insurance will not cover screening; therefore, they will not access primary care services [17, 18, 21, 22, 23]. However, it is important to note that being unable to afford healthcare is a barrier in countries without universal access to healthcare, such as the United States.

#### 3.6.3 External Facilitators of Help‐Seeking

Men report three approaches in which external facilitators can facilitate engagement in help‐seeking behaviour: improving access to healthcare services, improving awareness of abnormal LUTS and less invasive screening methods.

Men report that improving awareness of problems affecting LUTS will facilitate help‐seeking behaviour as men will have greater awareness of the potential severity of potential problems [27, 31]. Moreover, men from minority groups report that diverse health literature will facilitate help‐seeking as men from these groups will feel represented [22, 32].

Screening methods are important facilitators of help‐seeking, with men having an overall preference for less invasive screening methods [16, 22, 25, 26]. Screening methods are important facilitators of help‐seeking as Afro‐Caribbean men report a preference for PSA testing for PCa and report being more willing to engage in help‐seeking if the PSA test is the screening method of choice [25, 26]. Men have a preference for less invasive tests for CRC [16, 22]; however, some men report that financial incentives to screen will encourage some men to engage in screening for CRC [31].

## 4 Discussion

Using a narrative synthesis approach, this review aimed to explore the barriers and facilitators towards men face towards engaging in help‐seeking for abnormal LUTS. The data in the systematic review revealed a combination of factors, including a lack of awareness of abnormal LUTS, a lack of access to healthcare settings, taboos and cultural stigma over screening methods, poor relationships with HCPs and the fear of emasculation when screening. The reviewed papers consisted of men from an array of ages, cultures and countries. Men from minority groups reported greater barriers facing engagement in help‐seeking for abnormal LUTS, with some barriers more prominent across different conditions affecting LUTS, different cultures and countries. Nevertheless, men from minority groups also reported a greater amount of adjustments that can be made to facilitate their engagement in help‐seeking for abnormal LUTS.

Men are generally positive about attending primary care for abnormal LUTS; however, the thought of attending the doctor and the preceding screening procedures created psychological discomfort, fear and challenged perceptions of masculinity [12, 16, 17, 22, 25, 26, 27, 28, 29]. Men disliked the DRE and colonoscopy as screening methods for PCa and CRC as these procedures are perceived as emasculating, invasive and a violation of privacy [16, 17, 22, 26, 27, 29]. Moreover, among some Afro‐Caribbean men, the DRE is likened to a sexual act; further stigmatised by the taboo of homosexuality among Caribbean culture [26, 33]. To avoid the label of being “homosexual” attached to them, some Afro‐Caribbean men report the preference of having a female physician conduct the DRE [26]. However, “red flag” symptoms facilitated help‐seeking regardless of whether men liked the screening method or wanted to retain perceptions of masculinity. Normalising “going to the doctor” and “talking about one's health” may challenge men's attitudes that going to the doctors takes away one's masculinity as men will begin to perceive going to the doctor as a “normal” behaviour.

Men do not have trusting relationships with the healthcare system and healthcare professionals. Men across all groups and ages are reluctant to go to the doctor due to the shared perception that doctors do not have time for them [16, 17, 20]. However, men will attend the doctor once “red flag” symptoms are present ([12, 16, 17, 24]. The lack of trust in healthcare providers is greater among African American men due to the harrowing treatment of African Americans in recent American history [22, 27, 30, 31]. Moreover, African American men still do not report receiving equal treatment in healthcare in the United States [31]. Feelings of mistrust and reluctance to attend primary care are shared among black and Asian men in the United Kingdom for both physical and psychological symptoms [34]. Feelings of distrust are shared among ethnic minority groups, with the duty to mend these relationships landing on healthcare providers and policymakers.

Healthcare systems have a duty to re‐build trust with men from ethnic minorities. The lack of trust between minority ethnic groups and healthcare systems exacerbates health inequalities as some groups will avoid accessing primary care until symptoms or illness have progressed. Much of the lack of trust that ethnic minorities have with respective healthcare systems is due to historical malpractice towards ethnic minorities and people from ethnic minorities viewing the poor treatment that people from ethnic minorities often experience. The lack of trust between ethnic minorities and healthcare systems extends beyond abnormal LUTS in men, where they are evident across healthcare. For example, in the United Kingdom, children of Afro‐Caribbean and African descent are less likely to have a full course of the MMR vaccine as parents of Afro‐Caribbean and African descent have less trust in the healthcare system [35]. Men from ethnic minorities suggested that healthcare providers should work directly with men from minority groups to understand “why” men from some groups do not trust the healthcare system and work together to re‐build trust within the community, which will filter down into re‐building trust between ethnic minorities and healthcare systems [21, 30, 31, 32, 36].

Some men do not seek help for abnormal LUTS because there is a lack of awareness regarding abnormal LUTS. There is a lack of information available to the public to learn about symptoms affecting the lower urinary system [17, 20, 21, 25, 27, 28, 30, 31]. This is a significant barrier, yet men have no control over this barrier. Men cannot be expected to engage in help‐seeking for abnormal LUTS through the correct channels if they do not know what they are looking for or where they should go.

### 4.1 Clinical Implications

Healthcare providers and government policy should focus on re‐building relationships with minority groups through listening to suggestions made by the community to strengthen trust and reduce health disparities. Healthcare providers and policy makers have a duty to improve the awareness of men's health problems and de‐trivialise men's health. Improving disadvantaged men's access to healthcare services will incentivise men to access primary care for abnormal LUTS. Moreover, the implementation of universal healthcare will remove the financial barrier faced by millions of men across the world as healthcare will be free at the point of access [16, 20, 21, 22]. Moreover, strategies should be implemented to target higher risk groups so that help‐seeking for abnormal LUTS will become a “normal” behaviour.

Guidance and support for healthcare providers in the form of four recommendations for inclusive interventions may help to address the problem for men from ethnic minority groups. (1) A step to increase inclusivity is to improve the health literature to show that men from ethnic minority groups are represented. Representative health literature will encourage the de‐stigmatisation of abnormal LUTS as men from all groups will see people like them seeking help from the doctor for similar problems. (2) Secondly, healthcare professionals should demonstrate cultural sensitivity by spending more time listening to patients from minority groups, making them feel part of the discussion in the consultation. Men from ethnic minorities want healthcare providers to listen to their suggestions and want community outreach programmes. (3) Healthcare providers working with the community should also tackle another belief, the negative beliefs held towards the healthcare system as members of the community will speak to healthcare professionals on a personal level, therefore challenging the perceived authoritative stature of healthcare professionals [37]. (4) A fourth recommendation is that healthcare professionals should also be aided with extra training and guidance to improve their cultural sensitivity to improve the consultation experience for patients of ethnic minority groups.

### 4.2 Limitations of This Study

This review may have had a language bias as non‐English studies were excluded. This exclusion may result in the review having a lack of cultural depth. However, both English and American English variations of spellings were used to yield more papers from searches. Another limitation of this systematic review is that some of the sample populations of the studies included in the review were relatively small. The small sample sizes question the generalisability of the papers included in the study.

## 5 Conclusion

It is evident that men face a multitude of challenges when determining whether they will engage in help‐seeking for abnormal LUTS. The key findings from this review indicate that men lack an awareness of abnormal LUTS, men from ethnic minorities are distrusting of healthcare professionals, but men across all groups fear emasculation from screening and hold stigma and negative cultural views towards screening methods. Younger men tend to adhere to traditional gender stereotypes and appear to delay help‐seeking to appear masculine and macho. Our work suggests that healthcare policy should increase awareness of men's health problems, particularly problems affecting the lower urinary tract and rectum. Healthcare providers should also work with minority groups to reduce stigmatising beliefs held towards abnormal LUTS and work to re‐build relationships with minority groups.

## Author Contributions

**Stephen McIntosh:** conceptualization (lead), data curation (lead), formal analysis (lead), funding acquisition (equal), investigation (lead), methodology (lead), project administration (lead), resources (equal), software (equal), supervision (equal), validation (equal), visualization (lead), writing – original draft (lead), writing – review and editing (equal). **Bethany Harries:** methodology (equal), validation (equal). **Matthew Perry:** supervision (supporting), writing – review and editing (equal). **Mark Cropley:** supervision (supporting), writing – review and editing (equal). **Bridget Dibb:** conceptualization (supporting), data curation (supporting), funding acquisition (equal), investigation (supporting), methodology (supporting), project administration (supporting), supervision (lead), validation (supporting), writing – original draft (equal), writing – review and editing (equal).

## Conflicts of Interest

The authors declare no conflicts of interest.

## Supporting information


**Appendix S1:** cam471214‐sup‐0001‐AppendixS1.docx.

## Data Availability

Data sharing is not applicable to this article as no new data were created or analyzed in this study.

## References

[1] D. E. Irwin , Z. S. Kopp , B. Agatep , I. Milsom , and P. Abrams , “Worldwide Prevalence Estimates of Lower Urinary Tract Symptoms, Overactive Bladder, Urinary Incontinence and Bladder Outlet Obstruction,” BJU International 108, no. 7 (2011): 1132–1138, 10.1111/j.1464-410x.2010.09993.x. 21231991

[2] P. Rawla , T. Sunkara , and A. Barsouk , “Epidemiology of Colorectal Cancer: Incidence, Mortality, Survival, and Risk Factors,” Gastroenterology Review 14, no. 2 (2018): 89–103, 10.5114/pg.2018.81072. PMC6791134 31616522

[3] D. E. Irwin , I. Milsom , S. Hunskaar , et al., “Population‐Based Survey of Urinary Incontinence, Overactive Bladder, and Other Lower Urinary Tract Symptoms in Five Countries: Results of the EPIC Study,” European Urology 50, no. 6 (2006): 1306–1315, 10.1016/j.eururo.2006.09.019. 17049716

[4] Y. Xi and P. Xu , “Global Colorectal Cancer Burden in 2020 and Projections to 2040,” Translational Oncology 14, no. 10 (2021): 101174, 10.1016/j.tranon.2021.101174. 34243011 PMC8273208

[5] A. Y. Zhang and X. Xu , “Prevalence, Burden, and Treatment of Lower Urinary Tract Symptoms in Men Aged 50 and Older: A Systematic Review of the Literature,” SAGE Open Nursing 4 (2018): 2377960818811773, 10.1177/2377960818811773. 33415211 PMC7774430

[6] K. T. Rew , R. C. Langan , M. Hadj‐Moussa , and J. J. Heidelbaugh , “Men's Health: Scrotal and Testicular Conditions,” FP Essentials 503 (2021): 23–27. 33856180

[7] A. E. Foxx‐Orenstein , S. B. Umar , and M. D. Crowell , “Common Anorectal Disorders,” Gastroenterology & Hepatology 10, no. 5 (2014): 294–301. 24987313 PMC4076876

[8] J. A. Garcia , J. Crocker , and J. F. Wyman , “Breaking the Cycle of Stigmatization,” Journal of Wound, Ostomy, and Continence Nursing 32, no. 1 (2005): 38–52, 10.1097/00152192-200501000-00009. 15718956

[9] J. W. Griffith , E. E. Messersmith , B. W. Gillespie , et al., “Reasons for Seeking Clinical Care for Lower Urinary Tract Symptoms: A Mixed Methods Study,” Journal of Urology 199, no. 2 (2018): 528–535, 10.1016/j.juro.2017.07.067. 28734864 PMC5775934

[10] Z. Xu , F. Huang , M. Kösters , et al., “Effectiveness of Interventions to Promote Help‐Seeking for Mental Health Problems: Systematic Review and Meta‐Analysis,” Psychological Medicine 48, no. 16 (2018): 2658–2667, 10.1017/s0033291718001265. 29852885

[11] J. Sausville and M. Naslund , “Benign Prostatic Hyperplasia and Prostate Cancer: An Overview for Primary Care Physicians,” International Journal of Clinical Practice 64, no. 13 (2010): 1740–1745, 10.1111/j.1742-1241.2010.02534.x. 21070524

[12] A. Rubach , K. Balasubramaniam , M. M. Storsveen , S. Elnegaard , and D. E. Jarbøl , “Healthcare‐Seeking with Bothersome Lower Urinary Tract Symptoms Among Men in the Danish Population: The Impact of Lifestyle and Socioeconomic Status,” Scandinavian Journal of Primary Health Care 37, no. 2 (2019): 155–164, 10.1080/02813432.2019.1608412. 31056998 PMC6567136

[13] M. J. Page , J. E. McKenzie , P. M. Bossuyt , et al., “The PRISMA 2020 Statement: An Updated Guideline for Reporting Systematic Reviews,” BMJ (Clinical Research Ed.) 372 (2020): n71, 10.1136/bmj.n71. PMC8005924 33782057

[14] Q. N. Hong , P. Pluye , S. Fàbregues , et al., “Mixed Methods Appraisal Tool (MMAT) Version 2018 User Guide,” (2018), http://mixedmethodsappraisaltoolpublic.pbworks.com/w/file/fetch/127916259/MMAT_2018_criteria‐manual_2018‐08‐01_ENG.pdf.

[15] J. Popay , H. Roberts , A. Sowden , et al., “Guidance on the Conduct of Narrative Synthesis in Systematic Reviews. A Product From the ESRC Methods Programme. Version 1,” *Semantic Scholar*, (2006), 10.13140/2.1.1018.4643.

[16] M. Alsayid , N. M. Tlimat , C. Spigner , and C. Dimaano , “Perceptions of Colorectal Cancer Screening in the Arab American Community: A Pilot Study,” Primary Health Care Research & Development 20 (2019): e90, 10.1017/s1463423619000161. 32799969 PMC6609968

[17] F. A. Conde , W. Landier , D. Ishida , R. Bell , C. F. Cuaresma , and J. Misola , “Barriers and Facilitators of Prostate Cancer Screening Among Filipino Men in Hawaii,” Oncology Nursing Forum 38, no. 2 (2011): 227–233, 10.1188/11.onf.227-233. 21356660 PMC3662804

[18] O. U. Enaworu and R. Khutan , “Factors Influencing Nigerian Men's Decision to Undergo Prostate Specific Antigen Testing,” African Health Sciences 16, no. 2 (2016): 524–532, 10.4314/ahs.v16i2.21. 27605968 PMC4994573

[19] K. A. Ettridge , J. A. Bowden , S. K. Chambers , et al., ““Prostate Cancer Is Far More Hidden…”: Perceptions of Stigma, Social Isolation and Help‐Seeking Among Men With Prostate Cancer,” European Journal of Cancer Care 27, no. 2 (2018): e12790, 10.1111/ecc.12790. 29112317

[20] E. F. Ezenwankwo , C. A. Oladoyimbo , H. M. Dogo , et al., “Factors Influencing Help‐Seeking Behavior in Men With Symptoms of Prostate Cancer: A Qualitative Study Using an Ecological Perspective,” Cancer Investigation 39, no. 6–7 (2021): 529–538, 10.1080/07357907.2021.1933009. 34014791

[21] I. T. Forrester‐Anderson , “Prostate Cancer Screening Perceptions, Knowledge and Behaviours Among African American Men: Focus Group Findings,” Journal of Health Care for the Poor and Underserved 16, no. 4 (2005): 22–30, 10.1353/hpu.2005.0063. 16327094

[22] D. C. Fyffe , S. V. Hudson , J. K. Fagan , and D. R. Brown , “Knowledge and Barriers Related to Prostate and Colorectal Cancer Prevention in Underserved Black Men,” Journal of the National Medical Association 100, no. 10 (2008): 1161–1167, 10.1016/s0027-9684(15)31478-4. 18942277

[23] W. Hannöver , D. Köpke , and H.‐J. Hannich , “Perceived Barriers to Prostate Cancer Screenings Among Middle‐Aged Men in North‐Eastern Germany,” Public Health Nursing 27, no. 6 (2010): 504–512, 10.1111/j.1525-1446.2010.00889.x. 21087303

[24] M. King‐Okoye , A. Arber , and S. Faithfull , “Beliefs That Contribute to Delays in Diagnosis of Prostate Cancer Among Afro‐Caribbean Men in Trinidad and Tobago,” Psycho‐Oncology 28, no. 6 (2019): 1321–1327, 10.1002/pon.5085. 30953381 PMC6617795

[25] L. Medina‐Perucha , O. Yousaf , M. S. Hunter , and E. A. Grunfeld , “Barriers to Medical Help‐Seeking Among Older Men With Prostate Cancer,” Journal of Psychosocial Oncology 35, no. 5 (2017): 531–543, 10.1080/07347332.2017.1312661. 28368770

[26] O. N. Ocho and J. Green , “Perception of Prostate Screening Services Among Men in Trinidad and Tobago,” Sexuality Research & Social Policy 10, no. 3 (2013): 186–192, 10.1007/s13178-013-0118-5.

[27] J. S. Oliver , “Attitudes and Beliefs About Prostate Cancer and Screening Among Rural African American Men,” Journal of Cultural Diversity 14, no. 2 (2007): 74–80. 19175247

[28] M. M. Saab , M. Davoren , A. Murphy , et al., “Promoting Men's Awareness, Self‐Examination, and Help‐Seeking for Testicular Disorders: A Systematic Review of Interventions,” HRB Open Research 1 (2018): 1–16, 10.12688/hrbopenres.12837.2. PMC6973532 32002508

[29] S. Seymour‐Smith , D. Brown , G. Cosma , N. Shopland , S. Battersby , and A. Burton , ““Our People Has Got to Come to Terms With That”: Changing Perceptions of the Digital Rectal Examination as a Barrier to Prostate Cancer Diagnosis in African‐Caribbean Men,” Psycho‐Oncology 25, no. 10 (2016): 1183–1190, 10.1002/pon.4219. 27423059

[30] N. Shungu and K. R. Sterba , “Barriers and Facilitators to Informed Decision‐Making About Prostate Cancer Screening Among Black Men,” Journal of the American Board of Family Medicine 34, no. 5 (2021): 925–936, 10.3122/jabfm.2021.05.210149. 34535518

[31] V. Earl , D. Beasley , C. Ye , et al., “Barriers and Facilitators to Colorectal Cancer Screening in African American Men,” Digestive Diseases and Sciences 67 (2021): 463–472, 10.1007/s10620-021-06960-0. 33811563

[32] V. D. Woods , S. B. Montgomery , J. C. Belliard , J. Ramírez‐Johnson , and C. M. Wilson , “Culture, Black Men, and Prostate Cancer: What Is Reality?,” Cancer Control 11, no. 6 (2004): 388–396, 10.1177/107327480401100606. 15625526 PMC4654412

[33] E. J. Beck , K. Espinosa , T. Ash , et al., “Attitudes Towards Homosexuals in Seven Caribbean Countries: Implications for an Effective HIV Response,” AIDS Care 29, no. 12 (2017): 1557–1566, 10.1080/09540121.2017.1316355. 28438027

[34] M. S. Razai , T. Osama , D. G. J. McKechnie , and A. Majeed , “Covid‐19 Vaccine Hesitancy Among Ethnic Minority Groups,” BMJ (Clinical Research Ed.) 372, no. 8283 (2021): n513, 10.1136/bmj.n513. 33637577

[35] C. X. Zhang , C. Bankhead , M. A. Quigley , C. H. Kwok , and C. Carson , “Ethnic Inequities in Routine Childhood Vaccinations in England 2006‐2021: An Observational Cohort Study Using Electronic Health Records,” EClinicalMedicine 65 (2023): 102281, 10.1016/j.eclinm.2023.102281. 37965428 PMC10641103

[36] D. C. Fyffe , S. V. Hudson , J. K. Fagan , and D. R. Brown , “Knowledge and Barriers Related to Prostate and Colorectal Cancer Prevention in Underserved Black Men,” Journal of the National Medical Association 100, no. 10 (2008): 1161–1167, 10.1016/s0027-9684(15)31478-4. 18942277

[37] W. Sim , W. H. Lim , C. H. Ng , et al., “The Perspectives of Health Professionals and Patients on Racism in Healthcare: A Qualitative Systematic Review,” PLoS One 16, no. 8 (2021): e0255936, 10.1371/journal.pone.0255936. 34464395 PMC8407537

