# Supplementary material for: Barriers & Facilitators to Help‐Seeking Behaviour for Abnormal Lower Urinary Tract Symptoms in Men: Systematic Review
Source: Cancer Med. 2025 Sep 12;14(18):e71214. doi: 10.1002/cam4.71214 (PMC12427358; doi:10.1002/cam4.71214)

**Appendix**

**Appendix S1. Search Terms**

**Concept 1: Barriers & Facilitators:**

barrier* OR facilitator* OR utili?ation* OR support* OR access*

**Concept 2: Help-Seeking:**

“Health knowledge” OR “health attitude” OR “health promotion” OR “help-seeking” OR “help seeking” OR “help-seeking behaviour” OR attitude* OR "Health Knowledge, Attitudes, Practice"[Mesh]

**Concept 3: Abnormal LUTS Symptoms:**

“urinary problems” OR “genital problems” OR “genital changes” OR “lower urinary tract symptoms” OR luts OR "urinary hesitancy" OR "frequent urination" OR "urinary problems" OR "genital problems" OR "mens health" OR "male health" OR "genitourinary problems" OR "urinary disorders" OR "urological disorders" OR (((("urogenital neoplasms"[MeSH Terms]) OR "kidney neoplasms"[MeSH Terms]) OR "testicular neoplasms"[MeSH Terms]) OR "prostatic neoplasms"[MeSH Terms]) OR "Urinary bladder neoplasms"[MeSH Terms] OR "colorectal neoplasms"[MeSH Terms]

**Concept 4: Male**

Male OR man OR masculine* OR men

**Appendix S2. Quality Assessment: Mixed Method Appraisal Tool (MMAT)**

Attitudes and beliefs about prostate cancer and screening among rural African American men (Oliver, 2007)

| **Category** | **Methodical Quality Criteria** | Yes | No | Can’t tell | Comments |
| --- | --- | --- | --- | --- | --- |
| Screening Questions | S1. Are there clear research questions? |  |  |  |  |
|  | S2. Do the collected data allow to address the research question? |  |  |  |  |
| **Category of study design** | **Methodical Quality Criteria** | Yes | No | Can’t tell | Comments |
| Qualitative | 1.1 Is the qualitative approach appropriate to answer the research question? |  |  |  |  |
|  | 1.2 Are the qualitative data collection methods adequate to address the research question? |  |  |  |  |
|  | 1.3 Are the findings adequately derived from the data? |  |  |  |  |
|  | 1.4 Is the interpretation of results sufficiently substantiated by the data? |  |  |  |  |
|  | 1.5 Is there coherence between qualitative data sources, collection, analysis and interpretation |  |  |  |  |

Barriers and facilitators of prostate cancer screening in Filipino men in Hawai’i (Conde et al., 2011)

| **Category** | **Methodical Quality Criteria** | Yes | No | Can’t tell | Comments |
| --- | --- | --- | --- | --- | --- |
| Screening Questions | S1. Are there clear research questions? |  |  |  |  |
|  | S2. Do the collected data allow to address the research question? |  |  |  |  |
| **Category of study design** | **Methodical Quality Criteria** | Yes | No | Can’t tell | Comments |
| Qualitative | 1.1 Is the qualitative approach appropriate to answer the research question? |  |  |  |  |
|  | 1.2 Are the qualitative data collection methods adequate to address the research question? |  |  |  |  |
|  | 1.3 Are the findings adequately derived from the data? |  |  |  | Content Analysis |
|  | 1.4 Is the interpretation of results sufficiently substantiated by the data? |  |  |  |  |
|  | 1.5 Is there coherence between qualitative data sources, collection, analysis and interpretation |  |  |  |  |

Barriers and facilitators to colorectal cancer screening in African American men (Earl et al., 2022)

| **Category** | **Methodical Quality Criteria** | Yes | No | Can’t tell | Comments |
| --- | --- | --- | --- | --- | --- |
| Screening Questions | S1. Are there clear research questions? |  |  |  |  |
|  | S2. Do the collected data allow to address the research question? |  |  |  |  |
| **Category of study design** | **Methodical Quality Criteria** | Yes | No | Can’t tell | Comments |
| Mixed Methods | 5.1 Is there an adequate rationale for using a mixed methods design to address the research question? |  |  |  | Researchers state only study addressing this particular health issue |
|  | 5.2 Are the different components of the study effectively integrated to answer the research question? |  |  |  | Integration of methods only mentioned in discussion |
|  | 5.3 Are the outputs of the integration of qualitative and quantitative components adequately interpreted? |  |  |  |  |
|  | 5.4 Are divergencies and inconsistencies between quantitative and qualitative results adequately addressed? |  |  |  | States “substantiated in previous literature”. No explanation as to why |
|  | 5.5 Do the different components of the study adhere to the quality criteria of each tradition of the methods involved? |  |  |  |  |

Barriers and facilitators to informed decision making about prostate cancer screening among black men (Shungu et al., 2021)

| **Category** | **Methodical Quality Criteria** | Yes | No | Can’t tell | Comments |
| --- | --- | --- | --- | --- | --- |
| Screening Questions | S1. Are there clear research questions? |  |  |  |  |
|  | S2. Do the collected data allow to address the research question? |  |  |  |  |
| **Category of study design** | **Methodical Quality Criteria** | Yes | No | Can’t tell | Comments |
| Qualitative | 1.1 Is the qualitative approach appropriate to answer the research question? |  |  |  |  |
|  | 1.2 Are the qualitative data collection methods adequate to address the research question? |  |  |  |  |
|  | 1.3 Are the findings adequately derived from the data? |  |  |  |  |
|  | 1.4 Is the interpretation of results sufficiently substantiated by the data? |  |  |  |  |
|  | 1.5 Is there coherence between qualitative data sources, collection, analysis and interpretation |  |  |  |  |

Barriers to medical help-seeking among older men with prostate cancer (Medina-Perucha et al., 2017)

| **Category** | **Methodical Quality Criteria** | Yes | No | Can’t tell | Comments |
| --- | --- | --- | --- | --- | --- |
| Screening Questions | S1. Are there clear research questions? |  |  |  |  |
|  | S2. Do the collected data allow to address the research question? |  |  |  |  |
| **Category of study design** | **Methodical Quality Criteria** | Yes | No | Can’t tell | Comments |
| Qualitative | 1.1 Is the qualitative approach appropriate to answer the research question? |  |  |  |  |
|  | 1.2 Are the qualitative data collection methods adequate to address the research question? |  |  |  |  |
|  | 1.3 Are the findings adequately derived from the data? |  |  |  |  |
|  | 1.4 Is the interpretation of results sufficiently substantiated by the data? |  |  |  |  |
|  | 1.5 Is there coherence between qualitative data sources, collection, analysis and interpretation |  |  |  |  |

Beliefs that contribute to delays in diagnosis of prostate cancer among Afro-Caribbean men in Trinidad & Tobago (King-Okoye et al., 2019)

| **Category** | **Methodical Quality Criteria** | Yes | No | Can’t tell | Comments |
| --- | --- | --- | --- | --- | --- |
| Screening Questions | S1. Are there clear research questions? |  |  |  |  |
|  | S2. Do the collected data allow to address the research question? |  |  |  |  |
| **Category of study design** | **Methodical Quality Criteria** | Yes | No | Can’t tell | Comments |
| Qualitative | 1.1 Is the qualitative approach appropriate to answer the research question? |  |  |  |  |
|  | 1.2 Are the qualitative data collection methods adequate to address the research question? |  |  |  |  |
|  | 1.3 Are the findings adequately derived from the data? |  |  |  | Grounded theory |
|  | 1.4 Is the interpretation of results sufficiently substantiated by the data? |  |  |  |  |
|  | 1.5 Is there coherence between qualitative data sources, collection, analysis and interpretation |  |  |  |  |

Culture, black men, and prostate cancer: what is reality? (Woods et al., 2004)

| **Category** | **Methodical Quality Criteria** | Yes | No | Can’t tell | Comments |
| --- | --- | --- | --- | --- | --- |
| Screening Questions | S1. Are there clear research questions? |  |  |  |  |
|  | S2. Do the collected data allow to address the research question? |  |  |  |  |
| **Category of study design** | **Methodical Quality Criteria** | Yes | No | Can’t tell | Comments |
| Mixed Methods | 5.1 Is there an adequate rationale for using a mixed methods design to address the research question? |  |  |  | Does not give/ state rationale for conducting a mixed methods study |
|  | 5.2 Are the different components of the study effectively integrated to answer the research question? |  |  |  |  |
|  | 5.3 Are the outputs of the integration of qualitative and quantitative components adequately interpreted? |  |  |  |  |
|  | 5.4 Are divergencies and inconsistencies between quantitative and qualitative results adequately addressed? |  |  |  |  |
|  | 5.5 Do the different components of the study adhere to the quality criteria of each tradition of the methods involved? |  |  |  |  |

Exploring awareness and help-seeking intentions for testicular symptoms among heterosexual, gay and bisexual men in Ireland (Saab et al., 2017)

| **Category** | **Methodical Quality Criteria** | Yes | No | Can’t tell | Comments |
| --- | --- | --- | --- | --- | --- |
| Screening Questions | S1. Are there clear research questions? |  |  |  |  |
|  | S2. Do the collected data allow to address the research question? |  |  |  |  |
| **Category of study design** | **Methodical Quality Criteria** | Yes | No | Can’t tell | Comments |
| Qualitative | 1.1 Is the qualitative approach appropriate to answer the research question? |  |  |  |  |
|  | 1.2 Are the qualitative data collection methods adequate to address the research question? |  |  |  |  |
|  | 1.3 Are the findings adequately derived from the data? |  |  |  | Content analysis |
|  | 1.4 Is the interpretation of results sufficiently substantiated by the data? |  |  |  |  |
|  | 1.5 Is there coherence between qualitative data sources, collection, analysis and interpretation |  |  |  |  |

Factors influencing Nigerian men’s decision to undergo prostate specific antigen (PSA) testing (Enaworu et al., 2016)

| **Category** | **Methodical Quality Criteria** | Yes | No | Can’t tell | Comments |
| --- | --- | --- | --- | --- | --- |
| Screening Questions | S1. Are there clear research questions? |  |  |  |  |
|  | S2. Do the collected data allow to address the research question? |  |  |  |  |
| **Category of study design** | **Methodical Quality Criteria** | Yes | No | Can’t tell | Comments |
| Qualitative | 1.1 Is the qualitative approach appropriate to answer the research question? |  |  |  | Doesn’t state qualitative approach |
|  | 1.2 Are the qualitative data collection methods adequate to address the research question? |  |  |  |  |
|  | 1.3 Are the findings adequately derived from the data? |  |  |  | Does not state data analysis methods |
|  | 1.4 Is the interpretation of results sufficiently substantiated by the data? |  |  |  |  |
|  | 1.5 Is there coherence between qualitative data sources, collection, analysis and interpretation |  |  |  |  |

“Our people has got to come to terms with that”: Changing perceptions of the DRE as a barrier to PCa testing in Afro-Caribbean men (Seymour-Smith et al., 2016)

| **Category** | **Methodical Quality Criteria** | Yes | No | Can’t tell | Comments |
| --- | --- | --- | --- | --- | --- |
| Screening Questions | S1. Are there clear research questions? |  |  |  |  |
|  | S2. Do the collected data allow to address the research question? |  |  |  |  |
| **Category of study design** | **Methodical Quality Criteria** | Yes | No | Can’t tell | Comments |
| Qualitative | 1.1 Is the qualitative approach appropriate to answer the research question? |  |  |  |  |
|  | 1.2 Are the qualitative data collection methods adequate to address the research question? |  |  |  |  |
|  | 1.3 Are the findings adequately derived from the data? |  |  |  |  |
|  | 1.4 Is the interpretation of results sufficiently substantiated by the data? |  |  |  |  |
|  | 1.5 Is there coherence between qualitative data sources, collection, analysis and interpretation |  |  |  |  |

Perceived barriers to prostate cancer screenings among middle aged men in north-eastern Germany (Hannover et al., 2010)

| **Category** | **Methodical Quality Criteria** | Yes | No | Can’t tell | Comments |
| --- | --- | --- | --- | --- | --- |
| Screening Questions | S1. Are there clear research questions? |  |  |  |  |
|  | S2. Do the collected data allow to address the research question? |  |  |  |  |
| **Category of study design** | **Methodical Quality Criteria** | Yes | No | Can’t tell | Comments |
| Qualitative | 1.1 Is the qualitative approach appropriate to answer the research question? |  |  |  |  |
|  | 1.2 Are the qualitative data collection methods adequate to address the research question? |  |  |  |  |
|  | 1.3 Are the findings adequately derived from the data? |  |  |  | Did not state approach to coding. Only stated “content analytical procedures” |
|  | 1.4 Is the interpretation of results sufficiently substantiated by the data? |  |  |  | No use of quotes |
|  | 1.5 Is there coherence between qualitative data sources, collection, analysis and interpretation |  |  |  | Lack of use of quotes to interpret the data |

Perceptions of prostate cancer screening services among men in Trinidad & Tobago (Ocho et al., 2013)

| **Category** | **Methodical Quality Criteria** | Yes | No | Can’t tell | Comments |
| --- | --- | --- | --- | --- | --- |
| Screening Questions | S1. Are there clear research questions? |  |  |  |  |
|  | S2. Do the collected data allow to address the research question? |  |  |  |  |
| **Category of study design** | **Methodical Quality Criteria** | Yes | No | Can’t tell | Comments |
| Qualitative | 1.1 Is the qualitative approach appropriate to answer the research question? |  |  |  |  |
|  | 1.2 Are the qualitative data collection methods adequate to address the research question? |  |  |  |  |
|  | 1.3 Are the findings adequately derived from the data? |  |  |  |  |
|  | 1.4 Is the interpretation of results sufficiently substantiated by the data? |  |  |  |  |
|  | 1.5 Is there coherence between qualitative data sources, collection, analysis and interpretation |  |  |  |  |

Perceptions of colorectal cancer screening in the Arab American community (Alsayid et al., 2019)

| **Category** | **Methodical Quality Criteria** | Yes | No | Can’t tell | Comments |
| --- | --- | --- | --- | --- | --- |
| Screening Questions | S1. Are there clear research questions? |  |  |  |  |
|  | S2. Do the collected data allow to address the research question? |  |  |  |  |
| **Category of study design** | **Methodical Quality Criteria** | Yes | No | Can’t tell | Comments |
| Qualitative | 1.1 Is the qualitative approach appropriate to answer the research question? |  |  |  |  |
|  | 1.2 Are the qualitative data collection methods adequate to address the research question? |  |  |  |  |
|  | 1.3 Are the findings adequately derived from the data? |  |  |  |  |
|  | 1.4 Is the interpretation of results sufficiently substantiated by the data? |  |  |  |  |
|  | 1.5 Is there coherence between qualitative data sources, collection, analysis and interpretation |  |  |  |  |

Prostate cancer is far more hidden: perceptions of stigma, social isolation and help-seeking among men with prostate cancer (Ettridge et al., 2018)

| **Category** | **Methodical Quality Criteria** | Yes | No | Can’t tell | Comments |
| --- | --- | --- | --- | --- | --- |
| Screening Questions | S1. Are there clear research questions? |  |  |  |  |
|  | S2. Do the collected data allow to address the research question? |  |  |  |  |
| **Category of study design** | **Methodical Quality Criteria** | Yes | No | Can’t tell | Comments |
| Qualitative | 1.1 Is the qualitative approach appropriate to answer the research question? |  |  |  |  |
|  | 1.2 Are the qualitative data collection methods adequate to address the research question? |  |  |  |  |
|  | 1.3 Are the findings adequately derived from the data? |  |  |  | Thematic analysis |
|  | 1.4 Is the interpretation of results sufficiently substantiated by the data? |  |  |  |  |
|  | 1.5 Is there coherence between qualitative data sources, collection, analysis and interpretation |  |  |  |  |

Factors Influencing Help-Seeking behaviour in men with symptoms of Prostate Cancer: A Qualitative Study using an Ecological Perspective (Ezenwankwo et al., 2021)

| **Category** | **Methodical Quality Criteria** | Yes | No | Can’t tell | Comments |
| --- | --- | --- | --- | --- | --- |
| Screening Questions | S1. Are there clear research questions? |  |  |  |  |
|  | S2. Do the collected data allow to address the research question? |  |  |  |  |
| **Category of study design** | **Methodical Quality Criteria** | Yes | No | Can’t tell | Comments |
| Qualitative | 1.1 Is the qualitative approach appropriate to answer the research question? |  |  |  |  |
|  | 1.2 Are the qualitative data collection methods adequate to address the research question? |  |  |  |  |
|  | 1.3 Are the findings adequately derived from the data? |  |  |  |  |
|  | 1.4 Is the interpretation of results sufficiently substantiated by the data? |  |  |  |  |
|  | 1.5 Is there coherence between qualitative data sources, collection, analysis and interpretation |  |  |  |  |

Knowledge and Barriers related to Prostate and Colorectal Cancer Prevention in Undeserved Black Men (Fyffe et al., 2008)

| **Category** | **Methodical Quality Criteria** | Yes | No | Can’t tell | Comments |
| --- | --- | --- | --- | --- | --- |
| Screening Questions | S1. Are there clear research questions? |  |  |  |  |
|  | S2. Do the collected data allow to address the research question? |  |  |  |  |
| **Category of study design** | **Methodical Quality Criteria** | Yes | No | Can’t tell | Comments |
| Qualitative | 1.1 Is the qualitative approach appropriate to answer the research question? |  |  |  |  |
|  | 1.2 Are the qualitative data collection methods adequate to address the research question? |  |  |  |  |
|  | 1.3 Are the findings adequately derived from the data? |  |  |  |  |
|  | 1.4 Is the interpretation of results sufficiently substantiated by the data? |  |  |  |  |
|  | 1.5 Is there coherence between qualitative data sources, collection, analysis and interpretation |  |  |  |  |

Prostate cancer screening, perceptions, knowledge and behaviours among African American Men: Focus group findings (Forrester-Anderson, 2005)

| **Category** | **Methodical Quality Criteria** | Yes | No | Can’t tell | Comments |
| --- | --- | --- | --- | --- | --- |
| Screening Questions | S1. Are there clear research questions? |  |  |  |  |
|  | S2. Do the collected data allow to address the research question? |  |  |  |  |
| **Category of study design** | **Methodical Quality Criteria** | Yes | No | Can’t tell | Comments |
| Qualitative | 1.1 Is the qualitative approach appropriate to answer the research question? |  |  |  |  |
|  | 1.2 Are the qualitative data collection methods adequate to address the research question? |  |  |  |  |
|  | 1.3 Are the findings adequately derived from the data? |  |  |  |  |
|  | 1.4 Is the interpretation of results sufficiently substantiated by the data? |  |  |  |  |
|  | 1.5 Is there coherence between qualitative data sources, collection, analysis and interpretation |  |  |  |  |

**Appendix S3. Data Extraction**

| Title | Authors | Participants | Inclusion/Exclusion | Design & Data analysis | Findings | Conclusions | Themes |
| --- | --- | --- | --- | --- | --- | --- | --- |
| Attitudes and beliefs about prostate cancer screening among rural African Americans | Oliver, (2007) | (n = 9) African American men in rural Alabama, USA.  Aged between 43 – 72 years old.  Recruited through convenience sampling.  4/9 participants previously reported screening for PCa | Inclusion: Over 40 years old, English speaking, No personal history being diagnosed with PCa | Semi-structured interviews.  Interviews were recorded and transcribed verbatim.  Data was analysed using content analysis. | Analysis revealed the emergence of several themes.  *Disparity:* Disparity when accessing health care, African American men do not feel they are treated equally within the healthcare system.  *Lack of Understanding:* Participants knew little about PCa symptoms or screening methods for PCa.  *Traditions:* Past family practices influences the health patterns of participants.  *Mistrust of the system:* Historical events have created distrust of the healthcare system for African American men.  *Fear:* Participants expressed fear and concern over the screening methods  *Threat to manhood:* DRE is a violation, taking away manhood. | Remains distrust with the healthcare system due to historical events (*Tuskegee syphilis study*).  Perceptions and beliefs are key barriers to engagement in prostate cancer screening for this group.  Health care providers should make an effort to build a relationship with this group to aid the improvement of the existing barriers | Distrust in the healthcare system, lack of understanding of prostate cancer & screening methods |
| Barriers and facilitators of prostate cancer screening in Filipino men in Hawai’i | Conde et al., (2011) | (n = 20) Filipino men living in Hawai’i.  All participants were 40 or older, mean age 56 years old . | Exclusion: Self-reported or current diagnosis of PCa | Semi-structured interviews (focus groups).  Total of 5 focus groups consisting of 3 – 6 participants in each.  Interviews were recorded on audio tapes and transcribed verbatim.  Content analysis was performed manually by each member of the research team. | Differences between 1^st^ generation and 2^nd^ generation Filipino men.  Several barriers and facilitators towards PCa testing:  **Perceptions of Cancer:**  *Fatalism, dread & hopelessness:* 1^st^ generation Filipinos have fatalistic attitudes towards cancer 2^nd^ generation more knowledgeable and aware about treatment.  *Lack of Knowledge:* 1^st^ generation participants lacked knowledge of PCa.  *Misinformation*: Unsure of beliefs of PCa, belief it was caused by sexual promiscuity & lack of sex  *Risk factors:* caused by family history, diet, alcohol use & being overweight.  *Prevention beliefs:* Diet & exercise important for prevention.  **Barriers to PCa screening**  *Lack of awareness:* Poor awareness of PCa  *Reticence to help seeking:* Cultural barriers “Filipinos delay help seeking”.  *Help seeking delay:* cultural mindset to delay healthcare  *Fear:* Fear of being diagnosed & fear of death  *Financial issues:* Lack of money/ no health insurance  *Time constraints:* Both patient & HCP don’t have time – patient is busy and GP only has a few seconds  *Religious beliefs:* fatalism – God  *Embarrassment:* embarrassment to screen – DRE. Worse when nurse is female  **Facilitators:**  *Symptoms:* Men will go to the GP when they have symptoms (Filipino mentality)  *Concerns of Kidney Failure:* Fear of kidney failure – poor awareness – attribute LUTS to kidney  *HCP relationship:* Good relationship with HCPs with facilitate help-seeking  *HCP recommendations:* Recommendations from HCPs will encourage help-seeking  *Knowledge:* Knowledge of symptoms and testing | Key barriers to help-seeking: lack of awareness, lack of knowledge, negative beliefs, fears and only seeking delay when symptoms appear.  Similar behaviour to existing research in minority groups (African Americans & Hispanics).  Culturally relevant interventions are needed to address the barriers to prostate cancer screening, participation and misconceptions about the causes of prostate cancer. | **Barriers:** Fatalistic views, lack of knowledge of the causes of PCa and dysfunctional cultural beliefs towards cancer, external barriers as many men lack money.  **Facilitators:**  Improved relationships with clinicians and improving awareness |
| Barriers & Facilitators to colorectal cancer screening in African American Men | Earl et al., (2022) | *Total -* (n = 135) African American men from Atlanta metro area.  (n = 32) participants in semi-structured interviews.  (n = 103) participants in cross-sectional survey.  Participants recruited from primary care clinics and community centres | Not stated | *Part A:*  Semi-structured interviews.  Interviews guided by TPB and transtheoretical model.  Questionnaire featured 30 questions.  Interviews recorded and transcribed verbatim.  Interviews coded  *Part B:*  Cross-sectional study. Analysed using SPSS26.  Descriptive statistics on screening behaviour, knowledge, attitudes & barriers to screening, masculinity and health education.  Knowledge of screened vs non-screened using ANOVA & Chi-squared | **Part A:**  *Lack of knowledge of CRC screening:* 0/32 aware of all CRC screening methods. Most aware of colonoscopy. 1/32 sigmoidoscopy. 23/30 knew screening ages. Those who were screened had greater knowledge.  *Financial Barriers:* Incentive would encourage screening. Cost *not* a barrier. Screening would save money in long term.  *Cultural beliefs:* White people have better access to health care & culture promotes help-seeking. African American high comorbidity for diseases, fear of procedures (colonoscopy)  *Social influences:* Authority figures positive influences to screening. Greater knowledge in those who had relative screened. Half grew up in culture that promoted help-seeking. 2/14 knew somebody who screened (never screened group).  *Age:* 29/32 cared more about their health as they aged  *Masculinity:* Women weak & vulnerable. Men strong & macho – be responsible for family – motivator  **Part B:**  *Past & Future CRC screening:*  (46.1%) previous CRC test. (73%) reported test acceptable. (68.7%) would take test in the future. Preference: (31.7%) colonoscopy, (27.2%) stool test, (29.7%) unsure. (75%) interested in testing.  *Colon cancer knowledge:* scores ranged from 44.6% to 72.8%. Difference in screened vs never screened: (64.4 vs 60.2, F(1) = 0.87, *p* = .352).  *Barriers to CRC screening:* Barriers not perceived to be high. (48%) strongly agreed family provider did not recommend CRC screening, (48.5%) did not have family with CRC, (45.8%) did not know where to get test. (40.1%) strongly agreed provider did not recommend test or did not want to handle stool. (51%) strongly agreed did not want tube inserted in rectum.  *Masculinity:* Participants rated highly for masculinity domains: strength (*M* = 2.92), action approach (*M* = 2.88), optimistic capacity (*M* = 2.88), sexual impotence (*M* = 2.87) and family responsibilities (*M* = 2.86). Significant higher scoring in those who had been screened: (57.4 vs 86.9, F(1) = 18.82, *p <*0.01). α = 0.98.  *Persuasive CRC messages:* Most messages likely or most likely to persuade participants to get a test.  *Preferences for CRC education & programming:* Preference for reminder letters (55.3%) for CRC testing. (57.3%) preference for talking to providers, (46.6%) written materials and (44.7%) watching multimedia for education | Highlight the importance of family, masculinity, and community influences when promoting CRC screening in African American men.  Highlights disparities in CRC screening in African American men. | **Barriers:**  Lack knowledge of CRC, dysfunctional cultural beliefs, external barriers & machoism  **Facilitators:**  Preference for raising awareness, directly targeting men |
| Barriers and facilitators to informed decision making about prostate cancer among black men | Shungu et al., (2021) | (n = 21) self-identified black men.  Participants aged between 55 – 69 years old.  Participants recruited from F2F academic primary care clinic in South Carolina | Excluded: Men with history of PCa, undergoing cancer treatment, men with terminal illness or unable to give informed consent were excluded. | Semi-structured interviews.  5 focus groups consisting of 3 – 7 participants in each group.  Interviews recorded and transcribed verbatim.  Template analysis was used to analyse the data. Interviews were coded with themes then identified. | 3 Key themes were identified; external and intrinsic factors impacting informed decision making, beliefs impacting informed decision making  **External and intrinsic factors impacting IDM**  *Interpersonal influences:* Important individuals opinions and beliefs about health.  *Clinician communication:* Impact of clinicians on a man’s behaviour or understanding.  *Intrinsic factors:* Feeling of manhood encompassing the importance of strength, not wanting to burden others.  *Care avoidance:* Failure to care for oneself. Failure to seek help despite having agency.  *Motivated by racial disparities:* Black men motivated to seek additional health information to change behaviours  **Beliefs impacting IDM**  *Inadequate care from medical community:* Men perceive insufficient care or don’t believe medical community is correct.  *Perceived barriers:* Lack of access to screening, lack of awareness, lack of monetary barriers, fear of diagnosis, fear of screening procedure  *Perceived severity:* Generally thought PCa was terminal 🡪 influenced screening decisions  *Perceived benefits:* Generally believed screening can help save lives  **Target areas to facilitate IDM**  *Lack of knowledge:* participants felt black men lack knowledge of PCa screening to make IDM. Need more education.  *Takeaway from session:* New awareness screening is personal choice. Men need to consider own choices  *Areas of confusion:* Confused PCa screening with colon cancer & believed colonoscopy was for PCa. Only thought DRE was available. Thought side-effects from screening (ED) | African American men lack the knowledge of PCa to make informed decision making about screening options.  Clinicians play a vital role facilitating IDM through fostering conversations with African American men | **Barriers:**  Poor relationships & lack of trust with HCPs, masculinity, lack of knowledge of PCa, lack access to services and fear diagnosis  **Facilitators:**  Raise awareness of PCa |
| Barriers to medical help-seeking among older men with prostate cancer | Medina-Perucha et al., (2017) | (n = 20) British men. (n = 18) white men and (n = 2) black British. Participants ranged between 57 – 83 years old. Men from the MANCAN trial | Not reported | Semi-Structured interviews (qualitative). Interviews were analysed using thematic analysis. Interviews were in two parts: *Part A*: experiences on MANCAN trial. *Part B*: help-seeking and perceptions of help-seeking beliefs | Three main themes identified: male gender role, fear and embarrassment  **Male gender role:** main barrier to seeking help – restricted emotional expression, need for independence and control & viewing symptoms as minor. Minimising significance of medical symptoms led to avoidance of seeking help from HCPs.  **Fear:** Men were fearful of the health condition, medical procedure and screening and treatment progress  **Embarrassment:** Embarrassment of sexual related symptoms, medical examinations and communication with HCPs in the healthcare system. Medical exams and expression of sexually related symptoms were associated with a violation of men’s privacy and masculinity. Anticipation in such situations identified as a key barrier.  ***Other Barriers*:** Poor awareness of timely help-seeking, nonidentification of help-seeking delay and generational differences.  Those who previously experienced major health problems were aware of the importance of timely help-seeking. | Findings support the evidence of the traditional masculinity role as a barrier to help-seeking. Consistent with existing literature. | **Barriers:**  Perceptions of masculinity & machoism, fear & embarrassment of screening methods  **Facilitators:**  Previous health problems facilitates help-seeking |
| Beliefs that contribute to delays in diagnosis of prostate cancer among Afro-Caribbean men in Trinidad and Tobago | King-Okoye et al., (2019) | (n = 51) men from Trinidad and Tobago. Participants were recruited from urology and oncology centres (n = 2) in Trinidad and (n = 2) in Tobago. Participants were aged between 42 – 90 and diagnosed with PCa. | Not reported | Semi-structed interviews. Interviews were transcribed verbatim. Data was analysed using theoretical sampling and constant comparisons. Data was coded and themes were identified | Three themes were identified: Beliefs and meanings about PCa and normalisation of symptoms, taboos and sensitivity in the experience of bodily changes and use of herbs.  **Beliefs and meaning of PCa, normalisation of symptoms:**  Men normalised the symptoms of PCa eg, associated urine flow with age. Normalised symptoms of comorbidities rather than related them to prostate gland. Fatalism – Gods doing. Diagnosis a test from God, PCa a purpose for spiritual growth.  **Taboo and sensitivity in the experience of bodily changes:**  Sensitive nature of symptoms eg. Erectile problems made it difficult to engage in help-seeking. Contribution to help-seeking delay. Hiding bodily changes allows individuals to make sense of symptoms. Stoicism to manage symptoms. Use of OTC medication to further delay help-seeking through masking symptoms. Hiding symptoms due to perceptions that black men are stronger - Historical background. Unaware of the available tests/ screening. Uncomfortable with the DRE.  **Use of Herbs/ Self-management**  Keen self-management. Safer to approach by using natural remedies. Avoidance with healthcare services. Beliefs of side effects of medication were dangerous to the body. Lack of trust in doctors ability to diagnose. Use of traditional healers to help management of symptoms/ improve symptoms.  **Help-seeking**  Men who experienced “red flag” symptoms such as blood in urine and retention engaged in help-seeking in 1 – 7 days.  Joint pains, pins & needles, dribbling, straining took average 3 – 6 months to help seek. Some men took 2 years to seek help for nocturia, groin pain, fatigue and weight loss; some never reported ED. | Trinidad & Tobago men’s pre-diagnosis experiences of TCa are very important to understand the barriers to help-seeking.  Findings demonstrate taboo nature of symptoms and reluctance to help-seek. Highlight the effectiveness that community outreach  programmes may have to encourage TT men to engage in help-seeking | **Barriers:**  Dysfunctional cultural beliefs, taboo over symptoms and screening methods, lack knowledge of screening methods & PCa, alternatives to medical care  **Facilitators:**  “red flag symptoms” |
| Culture, Black Men, and Prostate Cancer: What is Reality? | Woods et al., (2004) | (n = 22) black men from Southern California. (n = 7) were physicians and (n = 2) were nurses. 2 focus groups were conducted with men from the target community. All participants were offered $15.00 incentive. | Non-Hispanic black American, at least 40 years old, no diagnosis of PCa and no evidence of mental illness | Mixed methods study; Qualitative and quantitative sections. | 5 Themes emerged on how cultural influences attitude, beliefs, and practices regarding decision-making about prostate cancer prevention. Themes were lack of knowledge, communication, social support, quality care and sexuality.  **Lack of knowledge:**  Men reported high knowledge of signs & symptoms of PCa. Men reported lack of knowledge to not having culturally appropriate health information & messages (*White men usually on literature in wating rooms).* Health care providers did not readily discuss prostate cancer information in a way that is understandable  **Complex Communication Issues:**  Poor communication with HCPs. HCPs did not fully explain benefits or risks of the PSA (47.4%) or not told why they were having the PSA (43.4%). (53.4%) indicated never been told they needed a PSA. Of those who had DRE, (58.3%) reported HCP did not say why they needed DRE and (63.2%) not told benefits and risks of DRE. Younger men reported least PSA testing  **Social Support:**  Black men provided support by talking to each other, older men were seen as role models. Significant others encouraged participation as they could learn more about PCa.  **Competence, Quality & Caring:**  (40%) felt the healthcare system is not designed to help African American men. Participants felt men did not screen as African American men were not aware they should be screened (79.5%), believed not at risk (78.4%), doctor never mentioned (66.7%), (62.8%) believed they were treated badly due to their race and (45.2%) believed they received poor quality health care due to race. (94%) believed early detection would improve chances of living longer.  **Sexuality:**  Participants concerned over impact of PCa on sexuality. (42.2%) concerned over ability to have erection, (44.5%) concerned over weak erections, (46.2%) concerned over erection too weak for vaginal penetration. Half reported uncertainty over being able to have an erection and (91.9%) reported functioning well sexually was important.  Lack of knowledge centred around black men not having basic information about prostate gland, prostate problems, consequences of PCa, | Lack of discussion over PCa screening and lack of culturally appropriate communication with healthcare providers has endangered trust, created fear, fostered disconnect and increased the likelihood of nonparticipation in prostate cancer screening in black men. | **Barriers:**  Lack of knowledge of PCa, poor HCP communication & lack of trust, challenges over masculinity & sexuality  **Facilitators:**  Improving trust with the healthcare system, social support |
| “Our people has got to come to terms with that”: Changing perceptions of the Digital Rectal Exam as a barrier to prostate caner diagnosis in African-Caribbean men | Seymour-Smith et al., (2016) | (n = 20) Afro-Caribbean men from the United Kingdom. (n = 10) diagnosed with PCa. (n = 10) without PCa. Opportunity sampling from community and PCa support group. Ages ranged from 30 to 83. | Not reported | Qualitative (semi-structured interviews). Interviews were transcribed and synthetic discursive approach was used to analyse the interviews. | Two themes were identified; Interpretive repertoire and positioning and accountability.  **Interpretative repertoire: homophobia and the DRE:**  DRE a key barrier to screening due to the perceived homosexuality of the procedure and the negative attitudes within the Afro-Caribbean community towards homosexuality. Perception the DRE is a sexual act. The Afro-Caribbean community has negative perceptions towards homosexuality hence the negative stigma to a procedure perceived to be homosexual. Socialisation that the rectum should not be touched within the community.  **Positioning and Accountability:**  Older Afro-Caribbean men have more negative attitudes towards the DRE. Not performing the DRE is a way to protect Afro-Caribbean masculinity. | Negative cultural stigma towards the DRE due to homophobic beliefs within the community. Societal norms have a big barrier to DRE performance in Afro-Caribbean men – Damages their perceived identity.  Health promotion interventions need to address the fear of homophobia and interventions are best designed with collaboration with the community | **Barriers:**  Dysfunctional cultural beliefs, sexualisation of screening methods, negative views towards screening methods |
| Perception of Prostate Screening Services among men in Trinidad & Tobago | Ocho & Green, (2013) | (n = 75) men from Trinidad & Tobago. Men were aged between 19 – 60 years old. Participants were recruited using “gatekeepers” in the community to ensure an equal distribution of participants to get an equal representation of participants across socio-demographic levels | Not Reported | Semi-Structured interviews (qualitative). Participants took part in 14 focus groups. Focus groups lasted between 45 to 90 minutes. Each focus group was recorded and transcribed verbatim. Data was analysed using a combination of inductive and deductive analysis | Three major themes emerged from the interviews: level of awareness, barriers and facilitators to accessing services.  **Level of Awareness:**  Respondents were aware of the importance of undergoing prostate exam as they grew older. Despite level of awareness, men were reluctant to engage in medical help-seeking or engaging with medical services. Asymptomatic men tend to “put it in the back burner”. Delay until they cannot bear symptoms anymore. Good awareness over the different tests available for the prostate exam.  **Barriers:**  More negative opinions towards the DRE. Typically described as invasive and associated with homophobia fears “homosexual act”. Physical discomfort of procedure not a barrier but interpretation of the experience. Homophobic anxiety towards the person conducting the exam – would it be a straight male conducting the DRE? Unwillingness to engage in help-seeking was perceived as threatening to masculine identity. Homosexual men also fearful of going to the doctor and disclosing their homosexuality for fear of being probed or discrimination.  **Facilitators:**  Men who’ve seen a loved one die from prostate cancer recognise the importance of seeking help “carry on tradition son – man”. Seeing a family member go through cancer likely to spur on screening.  PSA test is the preferred screening method due to the non-invasive nature and non-challenging to masculine identity.  Regards to DRE, men would prefer a female physician or a physician they don’t know. Feel, less uncomfortable and less threatening for their masculine identity. | Major barriers facing help-seeking are cultural beliefs with homophobic attitudes being a key barrier. Homophobic attitudes should be addressed in the long-term. Short term goals should focus on removing the association between the DRE and a sexual act.  Men have a preference for PSA testing rather than DRE as it is non-invasive and does not threaten masculinity | **Barriers:**  Lack of awareness of PCa, delay until “red flag symptoms”, negative views of screening methods, masculinity  **Facilitators:**  Previous experience with cancer, preference of PSA test, less threats to masculinity |
| Perceptions of Colorectal Cancer Screening in the Arab American Community: a Pilot study | Alsayid et al., (2019) | (n = 11) Arab American men from the San Francisco Bay area and Worcester, Massachusetts. Participants were aged between 50 – 75 years old. | Inclusion Criteria:  Aged 50 – 75 years old, born in an Arab country, identified as Arab and no active or prior history of colorectal cancer. Participants were recruited through adverts placed in Arab majority mosques and Mediterranean stores. Participants given short presentation about CRC after the interviews. | Semi-structured interviews (qualitative). Participants took part in 2 focus groups and 2 individual interviews. Interviews were recorded and transcribed verbatim. Grounded theory was used to analyse the data. Data was coded and themes were identified. | 6 Themes were identified and categorised in terms of barriers and facilitators: disbelief in modern medicine, concerns about the procedure, lack of communication with the physician (barriers), compliance and priority of health, access to healthcare services and awareness (facilitators).  **Disbelief in Modern Medicine:**  Disbelief in the word “cancer”, made up by pharmaceutical companies. No participants had regular CRC screening. Participants in favour of lifestyle changes  **Concerns over the procedure:**  Participants who had a colonoscopy reported uncomfortable and painful procedure. Reported the procedure is a key barrier to help-seeking for CRC. “would prefer if a new procedure would be discovered”.  **Lack of Communication with the Physician:**  Some participants reported their physicians did not provide them with appropriate care. GP appointments were very brief and irregular due to time restrictions. Participants without regular CRC screening stated GPs did not give them information about CRC screening methods or colonoscopy.  **Compliance and Priority of Health:**  Majority of participants agreed that compliance with the GPs recommendations was beneficial for their health. Agreement that preventative measures important.  **Access to Healthcare Services:**  Access to healthcare a major facilitator in help-seeking. Access to health care also introduced participants to CRC screening. Improvement of patient knowledge.  **Awareness:**  Need for regular screening is important. Some participants kept up with their CRC screening through discussions with family and friends whilst some learnt through physicians. Group generally believed that CRC is preventable and curable if found at an early stage. | Cultural beliefs contribute to Arab American men’s reluctance to engage in CRC screening. Preference for a ‘new’ screening method as colonoscopy perceived as a barrier to screening. Public health interventions to reduce disparities in help-seeking for Arab-American men for CRC | **Barriers:**  Lack of trust in HCPs & disbelief in medicine, dislike of screening methods for CRC, poor access to healthcare services  **Facilitators:**  Alternative screening method for CRC, improving awareness to CRC |
| “Prostate Cancer is far more hidden” Perceptions of Stigma, Social Isolation and Help-seeking among men with Prostate Cancer | Ettridge et al.., (2018) | (n = 20) men from Australia. Aged between 28 – 82 years old. | Inclusion Criteria: received either a diagnosis or treatment for PCa within the last 24 months, aged 18 years or older, proficient at English and capacity to provide informed consent. | Semi-Structured interviews (Qualitative). The first 3 interviews will be conducted in person and the final 17 conducted via telephone. Interviews were recorded and transcribed verbatim. Thematic analysis was used to analyse the transcripts. | Six main themes were identified: attributable causes of PCa, perceptions of stigma, prostate cancer and social isolation, overall impact of PCa, help-seeking (active-information and low emotional support) and unmet needs post treatment.  **Attributable Causes of Prostate Cancer:**  Participants regarded PCa as a “bad luck” cancer with not enough known to attribute a blame to. Differentiated PCa from other cancers like lung cancer where you could attribute a behaviour to causing the problem. Some participants reported a small degree of self-blame originating from past lifestyle or behaviour. One felt shame for not acting sooner and keeping diagnosis secret.  **Perceptions of Stigma:**  Older participants suggested there was no stigma due to their peers also having cancer/ health problems. Some suggestion stigma of PCa was self-inflicted illness, appeared to be related to having ‘cancer’ rather than PCa. Some stigma related to the perceptions of sex. Sense of embarrassment, awkwardness and shame towards PCa driven by treatment. Fear they may be judged as impotent. Some participants only became aware of other’s diagnoses once they disclosed theirs.  **Prostate Cancer and Social Isolation:**  Social isolation common, due to lack of available support, reluctance to talk, perceived withdrawal from others, limited by physical ans social consequences of treatment. Isolation due to lack of support to discuss diagnosis with. Others had withdrawn from them. Isolation due to not being able to socialise due to general health & treatment side effects. Social isolation more problematic for younger participants, those living along and with no family nearby.  **Overall impact of PCa:**  Prostate cancer had an emotional impact on participants (anxiety, loneliness, anger, questioning, fear of dying and side-effects.). Feeling of burdening partners, sexual dysfunction adverse effect on relationships. Treatment -loss of manhood appeared greater among younger participants. Diagnosed with a disease associated with “older age” challenged sense of mortality. Some accepted health issues are a part of ageing, some acceptance of sexual dysfunction (mostly with accepting partners).  **Help Seeking:**  Participants actively sought information for various sources: HCPs, websites, friends, nurses, written information and support groups. Typically did not seek formal (Psychologist) emotional support – most unaware option was available. Most sought help from informal sources (friends etc). Support groups an important source for some participants. However, some thought information was too subjective or not relevant. Some participants unwilling to talk to anyone - anticipated awkwardness, need to cope autonomously, not wanting to burden others, not wanting sympathy.  **Unmet need Post Treatment:**  Commonly shared acknowledgement of inadequate information, support or care following treatment from a physical or emotional perspective. Some dissatisfied with level of assistance from urologists post treatment | Perceptions of stigma were present amongst participants. Relate to sexual organs, treatment side effects, challenges to masculinity and attribution of the cancer cause and self blame.  PCa had considerable emotional impact on participants lives; both psychological and practical.  Men were willing to seek information but not additional support. | **Barriers:**  Dysfunctional beliefs towards cancer, stigma of PCa, lack of support  **Facilitators:**  Improving social support |
| Factors influencing Nigerian men’s decision to undego prostate specific antigen testing | Enaworu & Khutan, (2016) | (n = 10) Nigerian men aged between 40 – 60 years old. | Not specified. [*men 40-60 age range informed by recommendation made by American Urological Assicoation*] | Semi-structured interviews. Interviews were recorded and transcribed verbatim. Interviews were analysed thematically and guided by steps proposed by Colaizzi, (1978). | 5 core themes were identified:  **Symptoms experienced:**  Symptoms participants experienced influenced decision to engage in PSA testing. Experiencing symptoms (eg. Frequent urination, incontinence and pains) facilitated help-seeking. Family support and age also encouraged help-seeking. Not in all participants a one only visited doctor after pain persisting for many months.  **Influence of Friends:**  Men learnt about prostate cancer from friends & family who had been diagnosed with prostate cancer. Watching family & friends suffer from prostate cancer encouraged help-seeking and improved awareness among men about prostate cancer.  **Older age & increased awareness:**  Increasing age of the participants facilitated help-seeking. “older” participants reported being more health conscious as they aged and more aware of possible health problems.  **Accessibility of testing services:**  Accessibility of services heavily influenced decisions to engage in help-seeking. Participant who worked at a hospital reported great accessibility to test. Other participants reported the lack of accessibility to test. This was shared and participants suggested that the lack of accessibility is a barrier for men to engage in PSA testing.  **Knowledge of PSA testing:**  Participants knowledge of PSA testing improved with age. Men reported having greater knowledge of prostate cancer as they aged as they become more health aware/ conscious. Men also reported they improved their knowledge on prostate cancer from reading magazines and participation in this study.  ***Other findings***  Important to engage in PSA testing to detect potential abnormalities. | Age is facilitator to help-seeking. As men aged they become more health aware and more health conscious – therefore, more likely to engage in help-seeking for prostate cancer. Social influences also very important as important others advising one to seek help, watching others suffer from prostate cancer helped participants learn about prostate cancer. Accessibility to screening also a key barrier & facilitator. Men with access can screen, however, men who report a lack of access find it difficult to engage in screening. | **Barriers:**  Poor knowledge of PCa, lack of access to services  **Facilitators:**  Social support from friends & family, increased age, improving access to services, improving knowledge of prostate cancer |
| Exploring awareness and help-seeking intentions for testicular symptoms among heterosexual, gay, and bisexual men in Ireland: A qualitative descriptive study | Saab et al., (2017) | (n) = 29 men aged between 18 – 50 from the Republic of Ireland. | Inclusion Criteria: Male, aged 18 – 50, self identifying as heterosexual, gay or bisexual and residing in the Republic of Ireland | Qualitative descriptive study. Semi structured interviews. Interviews were recorded and transcribed verbatim. Inductive content analysis was used to analyse the transcripts. | Two main themes were identified  **Awareness of testicular disorders and their screening**  This theme was comprised of two sub-themes: impediments to awareness and enablers for awareness  *Impediments to awareness:*  All the participants had heard of testicular cancer, but had poor knowledge about testicular cancer. Trivialisation of testicular symptoms is a barrier as symptoms are taken for granted. Men also reported a greater focus on women’s health problems. Trivialisation of sex education at school under-represented some topics. Not knowledgeable of genital area, thought scrotal raphe was a problem but it’s a normal part of anatomy.  *Enablers to awareness*  Number of men reported being awareness of testicular disorders due to prior information, previous clinical exam or family history. Several learnt about the TSE through watching television and celebrities (Lance Armstrong). Few endorsed performing the TSE. Being gay was an enabler to awareness – assumption gay men are more in tune with their body.  **Help seeking intentions for testicular symptoms**  *Barriers to help-seeking intentions*  Several participants reported delay due to not knowing what to look for when performing the TSE “finding a lump in a bag of lumps”. Symptom misappraisal – participants believing pain is being caused by something else. Fear & denial also a barrier. Also fear of being labelled a hypochondriac. Belief that the pain would just go away. Help-seeking delay due to embarrassment, not wanting the GP to see or touch the participants testicles. Maladaptive coping, (denial and avoidance) when the feeling of a symptom present. Male social norms – stoicism, machoism, masculinity, men have to keep up a personality and seeking help makes you appear weak. | Overall participants heard of testicular cancer but lack knowledge on aspects of its malignancy. Poor knowledge on performance of the testicular self exam. Men reported several structural barriers to awareness of testicular cancer. Men also reported several barriers that delay help-seeking. The key reason for delay and maladaptive coping is social & psychological barriers. Men also reported facilitators to help-seeking, these can be used to encourage men to engage in help-seeking | **Barriers:**  Poor knowledge & awareness of PCa, masculinity, poor & mistrust of HCPs  **Facilitators:**  Sexuality (gay men have more interest in health) |
|  |  |  |  |  | 3 Main themes were identified: intrapersonal factors, interpersonal factors & institutional factors.  **Intrapersonal factors**  Participants had complete ignorance to PCa prior to diagnosis and lacked knowledge and symptom awareness. Most men experienced symptoms but due to lack of knowledge, some participants lived with the symptoms for more than a year before seeking medical help. Due to lack of knowledge of PCa symptoms, attributed symptoms to aged related problems, diabetes, infections and hypertension and spiritual attacks that would resolve in time. Men went to seek medical help when the symptoms became unbearable  **Interpersonal Factors**  Lack of family history of PCa, non disclosure of symptoms, perception of symptoms by family/ friends and socioeconomic status. Most men reported no knowledge of family history of PCa. Loss of dignity to disclose symptoms to associates. However, men reported encouragement and support from friends to seek attention from a healthcare provider. Being sole breadwinner negatively impacted decisions to engage in help-seeking. Some advise to seek help from herbal doctors from partners.  **Institutional Factors**  Past medical experiences and dysfunctional social beliefs as barriers. Some men reported difficulty in scheduling appointments and previous surgical errors. Access to medical services due to location also acted as a barrier to attending medical help seeking. Social beliefs also a substantial barrier in Nigeria. Communities do not encourage open conversation of PCa. Some view disease as caused by witchcraft, being unfaithful to partners, impotent and diagnosis makes you unfit to lead or participate in religious activities or rituals. Fatalistic beliefs as cancer is generally perceived as incurable. |  |  |
|  |  |  |  |  | Four key themes emerged from the analysis:  **motivation for seeking screening:**  Some participants were refereed to screening but some were non-adherent – citing lack of physician follow up, adopting a passive role and misinterpreting the importance of screening. Participants who completed prostate and CRC screening described as unpleasant *but* cited the benefits of knowing the outcome of screening the catalyst to completing their screening. Belief the benefits outweighed the negatives of screening were a catalyst to screening in men. Family history of cancer also triggered intentions to screen.  **Fear associated with prostate cancer colorectal cancer screening**  Fear was recurring and emphasised as a serious barrier to screening. Black men’s fear due to historical malpractice (i.e., Tuskegee syphilis experiment), fear over the screening procedures for CRC, misconception that the only way to screen is through a DRE. Participants suggested black men are sensitive to the DRE as this may lead to questions over their sexuality and manhood, men suggested that black men shy away from said procedures due to stigma and embarrassment associated with DRE. Fear of going to the doctor in black men. Fear of receiving screening results as a deterrent to seeking help.  **Healthcare system barriers:**  Participants reported three factors that hindered prostate and CRC screening: ***doctor-patient relationship*:** Previous negative experiences with healthcare practitioners, men described healthcare providers as poor communicators, disrespectful/ insincere. These beliefs demonstrated by inconsistent treatment from HCPs and lack of humanism. Some men were confident in HCPs and trusted their recommendations.  ***Mistrust of HCPs*:** Some do not trust HCPs due to not trusting the level of training they have received (junior doctors/ trainees), men want to talk to doctors not trainees. ***Insurance coverage*:** Type of insurance coverage was a systemic barrier as health insurance influenced the type of care you would be recommended.  **Methods to increase help-seeking/ screening**  To increase screening participants recommended, disseminating educational material to increase knowledge in black men. Person who is disseminating the information is important, men recommended that black male cancer survivors would be the most effective “role model” to target this group. Peer-to peer outreach seen as an effective method. Also encourage HCPs to get involved in community outreach to improve relationships. Family structures important source of information to promote prostate and CRC screening.  Many highlighted the importance of teaching about cancer during adolescence, providing black men with important tools for the future. Some have fears about peer to peer support over being perceived negatively by peers. |  |  |
|  |  |  |  |  | Only 42% of participants had been screened for prostate cancer on an annual basis. Seven themes emerged from the focus groups.  **Lack of knowledge:**  Minority of men had heard of the DRE, most were squeamish about the DRE and most did not know that there was a blood test for prostate cancer. Most men have preference for the PSA test over the DRE. Men generally disappointed doctors had not discussed screening for prostate cancer and were not provided with much information.  **Negative facts of life, lifestyle characteristics and cultural beliefs:**  General belief amongst men that negative beliefs and practices among African American men contribute to prevalence of PCa in African-American men. Sexual inactivity in elderly men thought to cause PCa. Status says we are inferior and there is nothing we can do, so why try. Processed food and nutritional factors influence risk of PCa.  **Fear:**  Men are fearful and shy away from testing. Men were worried about losing sexual function and reported that it can cause painful ejaculation, prevents one from having sexual intercourse and affects sexual performance. Men also report being scared to know the truth and fear going to the doctor despite knowing that prostate cancer is a killer amongst African American men. Men are also fearful of having the DRE performed on them.  **Embarrassment:**  The participants reported being embarrassed of preforming the DRE. Negative connotations surrounding the DRE make men feel embarrassed. Men who have performed the DRE say that performing the test does not hurt but also does not make you smile. A lot of psychological preparation is needed before performing the DRE. Men also reported that men in general do not talk to other men about their health in the same way that women talk to other women about their health.  **Disgust of government:**  Participants expressed mistrust for both the government and medical professionals. Belief amongst some men that PCa has something to do with the government due to the high prevalence in African American men. Belief that government may add things into the food that is distributed to black communities which may causes cancer. Participants also expressed that the culture is setup against black men’s health and black people are exploited.  **Lack of access and availability of tests:**  Economic status plays a big role in screening. Many men did not get screened for PCa because the tests were expensive and did not have insurance coverage. Men who had previous screening history were more likely to have greater knowledge of the DRE and PSA tests.  **Positive beliefs and hopes:**  Men who were more knowledgeable about the disease, men who faced PCa or men who knew someone that faced PCa were more positive and hopeful about the benefits of screening. Sooner that it is discovered, the higher the chance of curing PCa. The more you learn the better off you will be. Men also suggested various strategies that can be utilised to improve knowledge and encourage screening. For example, starting men's health meetings at churches and talking more about men’s health |  |  |
| Factors Influencing Help-Seeking behaviour in men with symptoms of Prostate Cancer: A Qualitative Study using an Ecological Perspcecitve | Ezenwankwo et al., (2021) | (n) = 27 men aged between 54 and 84 years old, first 2 years after diagnosis of PCa (n) = 9 currently undergoing treatment and (n) = 18 completed active treatment. (n) = 22 completed basic education, (n) = 13 retired from the civil service. | Eligibility: Diagnosed with PCa no later than 2 years at time of interview. | Qualitative interpretative descriptive design (semi structured interviews). Findings reported using the consolidated criteria for reporting qualitative research guidelines (COREQ) | 3 Main themes were identified: intrapersonal factors, interpersonal factors & institutional factors.  **Intrapersonal factors**  Participants had complete ignorance to PCa prior to diagnosis and lacked knowledge and symptom awareness. Most men experienced symptoms but due to lack of knowledge, some participants lived with the symptoms for more than a year before seeking medical help. Due to lack of knowledge of PCa symptoms, attributed symptoms to aged related problems, diabetes, infections and hypertension and spiritual attacks that would resolve in time. Men went to seek medical help when the symptoms became unbearable  **Interpersonal Factors**  Lack of family history of PCa, non disclosure of symptoms, perception of symptoms by family/ friends and socioeconomic status. Most men reported no knowledge of family history of PCa. Loss of dignity to disclose symptoms to associates. However, men reported encouragement and support from friends to seek attention from a healthcare provider. Being sole breadwinner negatively impacted decisions to engage in help-seeking. Some advise to seek help from herbal doctors from partners.  **Institutional Factors**  Past medical experiences and dysfunctional social beliefs as barriers. Some men reported difficulty in scheduling appointments and previous surgical errors. Access to medical services due to location also acted as a barrier to attending medical help seeking. Social beliefs also a substantial barrier in Nigeria. Communities do not encourage open conversation of PCa. Some view disease as caused by witchcraft, being unfaithful to partners, impotent and diagnosis makes you unfit to lead or participate in religious activities or rituals. Fatalistic beliefs as cancer is generally  perceived as incurable. | Overall, participants have very poor knowledge and awareness of PCa. Men lack the personal risk that they face, don’t disclose symptoms, fearful of the embarrassment and stigmatisation surrounding PCa, past hospital/ medical experiences and dysfunctional societal beliefs are key barriers to help-seeking. Family and friends do play a key role in encouraging medical help-seeking, supporting a patients decision attend a healthcare setting. Multi-faceted problem; intrapersonal, interpersonal and institutional factors. | **Barriers:**  Lack of awareness, dysfunctional cultural beliefs, lack of family history, socioeconomic status, difficulty accessing healthcare services, cancer fatalism  **Facilitators:**  “Severe” symptoms |
| Knowledge and Barriers related to Prostate and Colorectal Cancer Prevention in Undeserved Black Men | Fyffe et al., (2008) | Focus group (n) = 24 black men aged 22 – 85 years old. Participants from New Jersey. (n) = 0 had history of cancer but 48% had family history of cancer. | Not stated | 2 sets of focus groups conducted in 2 local churches. Focus groups were audiotaped and transcribed verbatim. Qualitative analysis used an immersion/ crystallisation approach. | Four key themes emerged from the analysis:  **motivation for seeking screening:**  Some participants were refereed to screening but some were non-adherent – citing lack of physician follow up, adopting a passive role and misinterpreting the importance of screening. Participants who completed prostate and CRC screening described as unpleasant *but* cited the benefits of knowing the outcome of screening the catalyst to completing their screening. Belief the benefits outweighed the negatives of screening were a catalyst to screening in men. Family history of cancer also triggered intentions to screen.  **Fear associated with prostate cancer colorectal cancer screening**  Fear was recurring and emphasised as a serious barrier to screening. Black men’s fear due to historical malpractice (i.e., Tuskegee syphilis experiment), fear over the screening procedures for CRC, misconception that the only way to screen is through a DRE. Participants suggested black men are sensitive to the DRE as this may lead to questions over their sexuality and manhood, men suggested that black men shy away from said procedures due to stigma and embarrassment associated with DRE. Fear of going to the doctor in black men. Fear of receiving screening results as a deterrent to seeking help.  **Healthcare system barriers:**  Participants reported three factors that hindered prostate and CRC screening: ***doctor-patient relationship*:** Previous negative experiences with healthcare practitioners, men described healthcare providers as poor communicators, disrespectful/ insincere. These beliefs demonstrated by inconsistent treatment from HCPs and lack of humanism. Some men were confident in HCPs and trusted their recommendations.  ***Mistrust of HCPs*:** Some do not trust HCPs due to not trusting the level of training they have received (junior doctors/ trainees), men want to talk to doctors not trainees. ***Insurance coverage*:** Type of insurance coverage was a systemic barrier as health insurance influenced the type of care you would be recommended.  **Methods to increase help-seeking/ screening**  To increase screening participants recommended, disseminating educational material to increase knowledge in black men. Person who is disseminating the information is important, men recommended that black male cancer survivors would be the most effective “role model” to target this group. Peer-to peer outreach seen as an effective method. Also encourage HCPs to get involved in community outreach to improve relationships. Family structures important source of information to promote prostate and CRC screening.  Many highlighted the importance of teaching about cancer during adolescence, providing black men with important tools for the future. Some have fears about peer to peer support over being perceived negatively by peers. | Help-seeking and screening behaviours for PCa and CRC in underserved black men may be influenced by constructs of the HBM including; background, personal perceptions, self-efficacy and cues to action. Men demonstrated knowledge of where to collect cancer information but lack awareness over the procedural aspects of prostate and CRC screening. Awareness often confused and limited. Fear of cancer and screening highlighted as important barrier. Fears based on beliefs and misconceptions that screening will have negative impact on masculinity and sexuality. Poor relationship and lack of trust with healthcare providers due to historical malpractice, perceived as counter-intuitive to seek help from medical services. | **Barriers:**  Poor HCP communication, lack of trust in HCPs, DRE impact sexuality, embarrassment, insurance (socioeconomic status)  **Facilitators:**  Benefits of screening, family history, increase awareness, community outreach |
| Prostate Cancer Screening Perceptions, Knowledge and Behaviours among African American Men: Focus group findings | Forrester-Anderson, (2005) | (n = 104) participants across 14 focus groups. Focus groups ranged from 6 to 12 participants. Men were aged between 40 – 80 years old from the Baltimore Metro area | Inclusion & exclusion criteria mentioned but not specific. Inclusion and exclusion criteria based upon age, race and county of residence | 14 focus groups, each ranging from 6 to 12 participants. Trained African-American facilitators served as leaders and co-leaders of the focus groups. Sessions were recorded and transcribed verbatim. Analysed using grounded theory. | Only 42% of participants had been screened for prostate cancer on an annual basis. Seven themes emerged from the focus groups.  **Lack of knowledge:**  Minority of men had heard of the DRE, most were squeamish about the DRE and most did not know that there was a blood test for prostate cancer. Most men have preference for the PSA test over the DRE. Men generally disappointed doctors had not discussed screening for prostate cancer and were not provided with much information.  **Negative facts of life, lifestyle characteristics and cultural beliefs:**  General belief amongst men that negative beliefs and practices among African American men contribute to prevalence of PCa in African-American men. Sexual inactivity in elderly men thought to cause PCa. Status says we are inferior and there is nothing we can do, so why try. Processed food and nutritional factors influence risk of PCa.  **Fear:**  Men are fearful and shy away from testing. Men were worried about losing sexual function and reported that it can cause painful ejaculation, prevents one from having sexual intercourse and affects sexual performance. Men also report being scared to know the truth and fear going to the doctor despite knowing that prostate cancer is a killer amongst African American men. Men are also fearful of having the DRE performed on them.  **Embarrassment:**  The participants reported being embarrassed of preforming the DRE. Negative connotations surrounding the DRE make men feel embarrassed. Men who have performed the DRE say that performing the test does not hurt but also does not make you smile. A lot of psychological preparation is needed before performing the DRE. Men also reported that men in general do not talk to other men about their health in the same way that women talk to other women about their health.  **Disgust of government:**  Participants expressed mistrust for both the government and medical professionals. Belief amongst some men that PCa has something to do with the government due to the high prevalence in African American men. Belief that government may add things into the food that is distributed to black communities which may causes cancer. Participants also expressed that the culture is setup against black men’s health and black people are exploited.  **Lack of access and availability of tests:**  Economic status plays a big role in screening. Many men did not get screened for PCa because the tests were expensive and did not have insurance coverage. Men who had previous screening history were more likely to have greater knowledge of the DRE and PSA tests.  **Positive beliefs and hopes:**  Men who were more knowledgeable about the disease, men who faced PCa or men who knew someone that faced PCa were more positive and hopeful about the benefits of screening. Sooner that it is discovered, the higher the chance of curing PCa. The more you learn the better off you will be. Men also suggested various strategies that can be utilised to improve knowledge and encourage screening. For example, starting men's health meetings at churches and talking more about men’s health | Structural factors a key barrier to help-seeking such as mistrust of the healthcare system, the government and high proportion of African American men in poverty, being unable to afford medical insurance or treatment. Key features of the African American experience. Men believed that screening is beneficial but fail to attend screening and often delay screening until there is an urgent need to attend healthcare. Cultural beliefs and practices are also key barriers to delay in help-seeking. Older age associated with a loss of sexual activity and a fatalistic approach towards cancer. Men also are sceptical over performing the DRE but have a preference for the alternative, PSA test | **Barriers:**  Lack of knowledge, poor HCP communication, dysfunctional cultural beliefs, fear of screening, fear of diagnosis, embarrassment of DRE, government mistrust, socioeconomic status  **Facilitators:**  Increased knowledge of disease, previous screening, targeted community outreach |

**Figure 1.**

*PRISMA flow diagram*


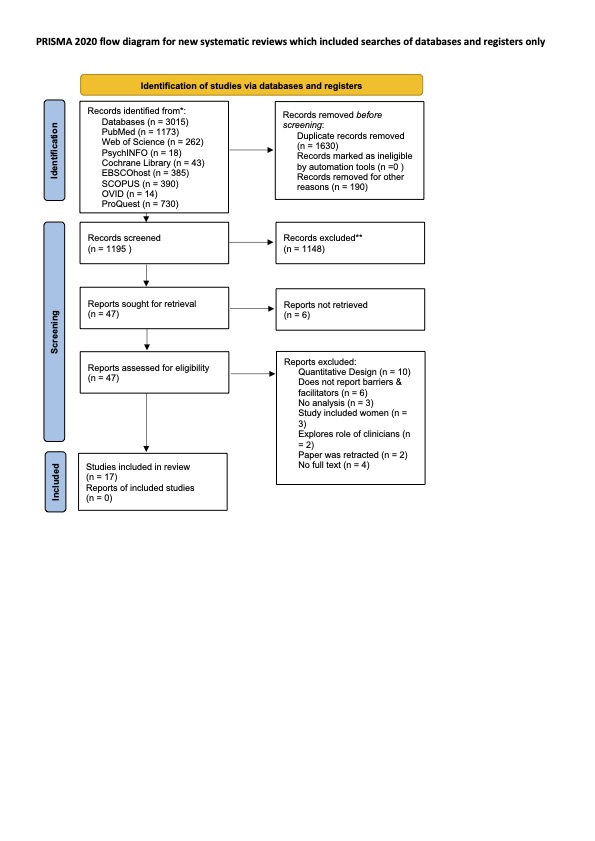

Supplement: Supplementary file 1 — Appendix S1: cam471214‐sup‐0001‐AppendixS1.docx. [file CAM4-14-e71214-s001.docx]
